# On the Stability of Complex Concentrated (CC)/High Entropy (HE) Solid Solutions and the Contamination with Oxygen of Solid Solutions in Refractory Metal Intermetallic Composites (RM(Nb)ICs) and Refractory Complex Concentrated Alloys (RCCAs)

**DOI:** 10.3390/ma15238479

**Published:** 2022-11-28

**Authors:** Panos Tsakiropoulos

**Affiliations:** Department of Materials Science and Engineering, Sir Robert Hadfield Building, The University of Sheffield, Mappin Street, Sheffield S1 3JD, UK; p.tsakiropoulos@sheffield.ac.uk

**Keywords:** high entropy alloys, complex concentrated alloys, refractory metal intermetallic composites, high entropy phases, complex concentrated phases, Nb silicide-based alloys, alloy design

## Abstract

In as-cast (AC) or heat-treated (HT) metallic ultra-high temperature materials often “conventional” and complex-concentrated (CC) or high-entropy (HE) solid solutions (sss) are observed. Refractory metal containing bcc sss also are contaminated with oxygen. This paper studied the stability of CC/HE Nb_ss_ and the contamination with oxygen of Nb_ss_ in RM(INb)ICs, RM(Nb)ICs/RCCAs and RM(Nb)ICs/RHEAs. “Conventional” and CC/HE Nb_ss_ were compared. “Conventional” Nb_ss_ can be Ti-rich only in AC alloys. Ti-rich Nb_ss_ is not observed in HT alloys. In B containing alloys the Ti-rich Nb_ss_ is usually CC/HE. The CC/HE Nb_ss_ is stable in HT alloys with simultaneous addition of Mo, W with Hf, Ge+Sn. The implications for alloy design of correlations between the parameter δ of “conventional” and CC/HE Nb_ss_ with the B or the Ge+Sn concentration in the Nb_ss_ and of relationships of other solutes with the B or Ge+Sn content are discussed. The CC/HE Nb_ss_ has low Δχ, VEC and Ω and high ΔS_mix_, |ΔH_mix_| and δ parameters, and is formed in alloys that have high entropy of mixing. These parameters are compared with those of single-phase bcc ss HEAs and differences in ΔH_mix_, δ, Δχ and Ω, and similarities in ΔS_mix_ and VEC are discussed. Relationships between the parameters of alloy and “conventional” Nb_ss_ also apply for CC/HE Nb_ss_. The parameters δ_ss_ and Ω_ss_, and VEC_ss_ and VEC_alloy_ can differentiate between types of alloying additions and their concentrations and are key regarding the formation or not of CC/HE Nb_ss_. After isothermal oxidation at a pest temperature (800 ^o^C/100 h) the contaminated with oxygen Nb_ss_ in the diffusion zone is CC/HE Nb_ss_, whereas the Nb_ss_ in the bulk can be “conventional” Nb_ss_ or CC/HE Nb_ss_. The parameters of “uncontaminated” and contaminated with oxygen sss are linked with linear relationships. There are correlations between the oxygen concentration in contaminated sss in the diffusion zone and the bulk of alloys with the parameters Δχ_Nbss_, δ_Nbss_ and VEC_Nbss_, the values of which increase with increasing oxygen concentration in the ss. The effects of contamination with oxygen of the near surface areas of a HT RM(Nb)IC with Al, Cr, Hf, Si, Sn, Ti and V additions and a high vol.% Nb_ss_ on the hardness and Young’s modulus of the Nb_ss_, and contributions to the hardness of the Nb_ss_ in B free or B containing alloys are discussed. The hardness and Young’s modulus of the bcc ss increased linearly with its oxygen concentration and the change in hardness and Young’s modulus due to contamination increased linearly with [O]^2/3^.

## 1. Introduction

The interdepended targets for performance and environmental impact of future aero engines could be met with materials that would allow high pressure turbines to operate at significantly higher than current temperatures. In other words, ultra-high temperature materials (UHTMs) with capabilities beyond those of Ni-based superalloys are needed [1]. UHTMs must meet property goals for fracture toughness, oxidation resistance and creep [2]. The fracture toughness property goal necessitates the new materials to show some degree of metallic behaviour to distinguish them from ceramic UHTMs [3]. Research and development work is in progress to find metallic UHTMs that can be used in structural engineering applications [2,3,4,5,6,7,8].

Metallic UHTMs depend on refractory metal (RM) additions and include RM intermetallic composites (ICs), i.e., RMICs, RM high entropy alloys (HEAs), i.e., RHEAs and RM complex concentrated alloys (CCAs), i.e., RCCAs. This classification is logically and pragmatically exhaustive. Not all RHEAs or RCCAs are RMICs, but some are. Moreover, not all RMICs are RHEAs or RCCAs, but some are. RMICs based on the Nb-Si system, i.e., RM(Nb)ICs or the Mo-Si system, i.e., RM(Mo)ICs are under development [3,8]. Some of the former are also high-entropy or complex concentrated alloys, i.e., RM(Nb)IC/RHEA or RM(Nb)IC/RCCA [3,9]. In this paper ceramic UHTMs and RM(Mo)ICs are not considered.

The RM(Nb)ICs, RM(Nb)ICs/RCCAs and RM(Nb)ICs/RHEAs are multiphase alloys with phases such as bcc solid solution(s), silicide(s), C14 Laves and A15 compounds, and other intermetallics [10,11,12,13]. These phases can be “conventional” phases or high entropy (HE) phases or complex concentrated (CC) (compositionally complex) phases [3,10,14]. HE or CC eutectics and/or HE or CC lamellar microstructures also can form in their microstructures [13,15,16]. The “conventional” phases can co-exist with the CC/HE phases in the as cast (AC) and/or heat treated (HT) conditions or after oxidation [13,14,17,18]. Phase transformations of CC intermetallics can generate unusual microstructures in RM(Nb)ICs [15]. HEAs and HE phases are those where the maximum and minimum concentrations of elements are not above or below, respectively, 35 and 5 at.%, whereas RCCAs and CC phases are those where the maximum and minimum concentrations of elements are above 35 at.% (up to about 40 at.%) and below 5 at.% [3,9,19].

The microstructures of RHEAs and RCCAs can be single phase or multiphase, namely solid solution(s) with/without intermetallics, for example M_5_Si_3_ silicides owing to Si addition (M = transition metal (TM) and/or RM) or Laves phases [19]. RMICs, RHEAs and RCCAs share the same alloying elements [3,9,14]. In the pairings RMIC-RHEA and RMIC-RCCA the two terms are mutually complementary (the same is the case for the pairings HEA-CCA, RHEA-RCCA, RM(Nb)IC-RM(Mo)IC). The development of RM(Nb)ICs is linked with the study of intermetallics and the development of intermetallic-based alloys (e.g., [1,20,21]), in contrast with the development of RHEAs and RCCAs that resulted from research on HEAs [19]. For these three categories of metallic UHTMs there is a significant volume of research [3,19]. Methods of preparation of metallic UHTMs are discussed in [2,3,19].

### 1.1. Alloy Design and the Alloy Design Methodology NICE

Groups of alloys (e.g., Ni-based superalloys for blade or disc applications in gas turbine engines) exhibit striking regularities [22]. Metallurgists who develop new alloys can have data that might not be directly intelligible as they stand and with relationships that are not immediately apparent [9]. Time and again, enthalpy and entropy of mixing, electronegativity, atomic size, electron-to-atom ratio and relationships based on these parameters provide an intermediate step to link the data, to weave them into a framework of understanding that is subtle and mathematical. Parameters based on the aforementioned thermo-physical and structure properties can reflect, albeit imperfectly, actually existing properties of alloys that help us uncover new things about alloys and their phases, sometimes things we never suspected, to uncover regularities and linkages and to establish relationships between different properties [9]. This has been demonstrated for rapidly solidified crystalline and amorphous alloys, bulk metallic glasses, HEAs and RM(Nb)ICs, for example [3,9,10,14,23,24,25,26,27]. Relationships between parameters of alloys and their phases, between the same parameters and properties of alloys and their phases have shown that there is an elegant simplicity that is underpinned by definite mathematical relationships that interweave each other to form via their interrelatedness and interdependent influences a subtle and harmonious methodology to process alloy design/selection through progressive goal-oriented approach [9,10,14,28,29,30,31]. This design methodology is known as NICE [10]. It was founded on data for RM(Nb)ICs [10] and has been expanded to cover RHEAs and RCCAs with Nb and Si addition [3,9,15,32,33,34,35]. The papers [28,29,30,31] dealt closely with questions that pertain to the alloying behaviour and properties of key phases in RM(Nb)ICs, RM(Nb)ICs/RCCAs and RM(Nb)ICs/RHEAs. In [9], a succinct account was given of the approach and aspirations upon which the said papers and [26] “converged” and were “unified” in NICE [10]. One could visualise this research as a “fruit producing tree”. The study in [26] forms the trunk, the “sprinkle of water” that “feeds its growth” is from [9] and new research, [28,29,30,31] are its “branches” and [10], i.e., NICE, is its “fruit”. Manifestations of the “juiciness” of this “fruit” are [13,14,16,17,18,32,33,34,35,36,37,38] and this paper.

As it will be demonstrated in this paper, NICE helps the alloy developer to find unexpected new relationships as the range of investigation of metallic UHTMs is expanded. NICE depends on high quality chemical analysis data for the calculation of parameters based on aforementioned properties, namely the parameters ΔH_mix_, ΔS_mix_, δ, Δχ, VEC and Ω, which are the same parameters used to study HEAs and CCAs [3,10,26,27,28,39,40,41]. With NICE, a material system suitable for application in high pressure turbine and comprising a metallic UHTM substrate plus metallic bond coat of an environmental coating of the bond coat/thermally grown oxide/ceramic top coat type can be designed [14].

**Figure 1:**
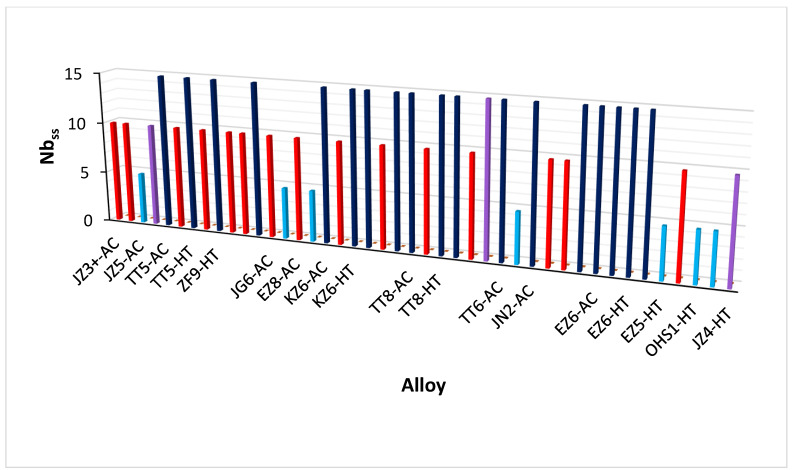
Nb_ss_ in RM(Nb)ICs and RM(Nb)ICs/RCCAs with nominal Si content 18 at.% and alloying elements Al, B, Cr, Ge, Hf, Mo, Nb, Sn, Ta, Ti, W: “conventional” Nb_ss_ (dark blue), CC/HE Nb_ss_ (red), (iii) Nb_ss_ with no Si (light purple) (iv) not stable Nb_ss_ (light blue). For presentation purposes the numbers 15, 10 and 5 have been assigned, respectively, to “conventional” Nb_ss_, CC/HE Nb_ss_ and not stable Nb_ss_. AC = as cast, HT = heat treated. For nominal alloy compositions and references see the Appendix A. RM(Nb)ICs/RCCAs the alloys JZ3+, JZ5, TT5, ZF9, JG6, EZ8, TT7, OHS1, JZ4. HE Nb_ss_ in TT4-AC.

Although metallic UHTMs can be complex, they are clearly not random. We observe regularities and patterns, and organise these into relationships which are used in NICE and give it predictive power [9,14,26,28]. For example, the boron containing RM(Nb)ICs and RM(Nb)ICs/RCCAs occupy a specific corner in the Δχ versus δ map or a specific area in the ΔH_mix_ versus Δχ map [3,14,26], oxidation resistant RM(Nb)ICs and RM(Nb)ICs/RCCAs have low VEC and high δ values [10,18,32,34,35,36,42]. In these metallic UHTMs the “behaviour” of one element is inextricably entangled with those of the others via the aforementioned parameters and the relationships that have been found between them, for example see Figures 12 and 16 in [10], Figures 12–18 in [15], Figures 1 and 2 in [29], Figures 1–6 in [30], Figures 1–11 in [31], Figures 12–14 in [33], Figures 9, 12 and 13 in [34], Figures 7–15 in [35], Figures 4 and 5 in [36], Figure 13 in [37], Figures 10 and 11 in [43] and Figures 8 and 9 in [44]. The available data give a realistic (workable, effective, consistent) account of how the alloying behaviour and properties of alloys and their phases are “determined (controlled)” by different groups of elements working in synergy in a metallic UHTM [3,9,10,14].

One way of expressing this “quality” of metallic UHTMs, meaning the regularities that they show, is to say that these materials have organised complexity. This organisation is captured by NICE, which focuses on the amount of information needed and its quality and value. Regularities are systematised into relationships [9,10,29,30,31]. Given a property goal, these relationships are used in NICE to calculate the chemical composition of an alloy, properties of which also can be computed [3,9,10,14,33,34,37,38,45]. Underlying the complexity of metallic UHTMs is the apparent simplicity of relationships that enable organised complexity to emerge. The organizational properties of these complex alloys are attributed to the relationships of parameters that reflect the specific nature of the alloys concerned. Regularities possess contingent features, meaning they depend upon something beyond themselves, for example, contamination by interstitials (see below in this section and Section 3.1) owing to interaction with the environment, and thus parameter values and relationships change (see Section 3 below).

The design/selection of new alloys is possible using NICE [10,14]. Design constraints pertaining to an alloy of interest can be traced to the wider alloying environment, for example see [33,34,37,38,45]. One of the main features of NICE is that the “affairs” of alloys cannot be separated from the “affairs” of phases and the parameters that describe alloying behaviour and properties of alloys and phases. It is a linkage that has profound implication for the design of metallic UHTMs [14].

NICE shows that metallic UHTMs must be understood holistically and that the properties of a metallic UHTM are comprehended by studying the alloying behaviour and properties of its constituent phases. In other words, NICE proposes two complementary ways of studying alloy development using both reductionist and holistic approaches. Akin to all alloys, a RM(Nb)IC, RHEA or RCCA is a physical system with a collection of atoms of different elements with similar or different concentrations and different levels of structure (meaning the different or similar structures of elements and of the phases such as solid solution(s) and intermetallic(s) that make up the alloy microstructure with a particular “architecture” (e.g., co-continuous solid solution(s)-intermetallic(s)), influenced by internal processes (e.g., solute partitioning) or the environment (e.g., contamination with interstitials) in which the alloy is produced and/or operates. For example, partitioning of solutes can result (i) to change in crystal structure (the case of Ti partitioning to Nb_5_Si_3_ and substituting Nb, thus causing a change in structure from tetragonal to hexagonal [46]) or (ii) formation of sub-grains in Nb_5_Si_3_ [47], while change in structure also can occur with contamination with interstitials (for example, the case of hexagonal instead of tetragonal Nb_5_Si_3_ stabilised in Nb-Si alloys with C contamination [48]).

In RM(Nb)ICs, RM(Nb)ICs/RCCAs or RM(Nb)ICs/RHEAs and single phase or multiphase RCCAs or RHEAs the solid solution(s) will be contaminated with oxygen, and the severity of contamination will differ, depending on alloying additions and their concentrations, and exposure conditions [13]. Alloying strategies might be able to counterbalance effects of interstitial contamination on properties. For example, grain-boundary segregation of oxygen caused room-temperature brittleness of the as cast (AC) single phase solid solution NbMoTaW RHEA. Alloying with B from 400 ppm (0.04 at.%) to 8000 ppm (0.8 at.%) offset this effect of O and improved the mechanical properties at room temperature. Both strength and plasticity were improved and reached maximum values at around 5000 ppm (0.5 at.%) B addition. Specifically, the plasticity increased from <2% to >10% and the fracture strength increased from 1211 MPa to 1780 MPa, respectively, for the base RHEA and the RHEA alloyed with 5000 ppm B. However, the plasticity of the said RHEA decreased with further increase in the B concentration [49]. Contamination with oxygen can have a strong effect of the near surface properties of phases and alloy [3]. This paper will show how NICE helps the alloy developer to understand the effect of contamination with oxygen on the properties of the bcc solid solution.

### 1.2. Aim of This Work

HE or CC phases can co-exist with “conventional” phases and can be stable in RM(Nb)ICs, RM(Nb)ICs/RCCAs or RM(Nb)ICs/RHEAs [10,14]. Phase transformations of CC silicides give new simple and/or complex microstructures the importance of which for the properties of alloys has not been studied or considered in modelling research, e.g., modelling of creep [50]. Are the CC or HE bcc solid solutions stable? Is their stability dependent on alloying additions, alloy condition (meaning AC or heat treated (HT)) and contamination with oxygen? Boron or Ge and Sn have a distinctive effect on the alloying behaviour and properties of the aforementioned materials [26,32,33,34,45] and the Nb_5_Si_3_ silicide [9,14,29]. Is the stability of CC/HE Nb_ss_ dependent on the presence of B or Ge and Sn in the alloy? Are there similarities regarding the dependence of other solute addition concentrations on the B or Ge+Sn content of solid solutions? How does the contamination of bcc Nb_ss_ with oxygen or alloying with boron affect its properties? The motivation for this paper was to provide answers to these questions.

I shall consider some of the possible permutations of available data between aforementioned parameters and between parameters and solutes under two major headings, namely “complex concentrated bcc solid solution” and “contamination of the bcc solid solution with oxygen”. There is a logic behind this approach in this paper, as I shall aim to show. The four solutes Ge, Sn, B and O will be a focus, and the latter two will be the point of reference when I shall discuss the hardness of the bcc solid solution. All four solutes are remarkably untypical in RHEAs and RCCAs studied to date (e.g., see [19]) even though they are essential additions in RM(Nb)ICs, RM(Nb)ICs/RCCAs and RM(Nb)ICs/RHEAs for balance of properties. The first three, Ge and Sn together and B on its own or in synergy with Ge or Sn can assist the alloy developer to obtain metallic UHTMs with a balance of properties by making use of the synergies of these three elements with Al, Cr, Hf, Si and Ti, as suggested by research on RM(Nb)ICs/RCCAs and RM(Nb)ICs/RHEAs, e.g., see [9,14,32,33,34,35,36]. Oxygen is a solute the presence of which cannot be avoided in UHTMs with RM additions owing to the sensitivity of RMs to interstitial contamination (e.g., see [3] and Section 3.1 below). The contamination with oxygen has profound implications for properties of phases (this will be demonstrated for the solid solution in this paper) that (a) should not be ignored in studies of processing-microstructure-property relationships in UHTMs, as discussed in [3,19], and (b) can be used to design specific microstructures to improve properties, for example see the “design and selection of Nb-Al-Si-Hf-Ti alloys” in [37,38].

The paper is consciously selective. It does not deal with CC/HE silicides, C14 Laves and A15 compounds, eutectics and lamellar microstructures and their contamination with oxygen. It is intended to open further questions about bcc solid solutions in metallic UHTMs and to suggest future research. It is not a system of polarities (opposite characteristics) (meaning “conventional”–CC/HE, contaminated –“uncontaminated” phase) that we have to deal with but an overlapping set of interrelationships (see below) and transformations [13,16], which are viewed in the context of metallic UHTM development and provide a useful route and compass for exploring the microstructures of these materials.

Given that the analysis of data for bcc solid solutions will be based on aforementioned parameters, the calculation of which requires high quality chemical analysis data [9,10,14], this paper concentrates only on the bcc solid solutions in RM(Nb)ICs, RM(Nb)ICs/RCCAs and RM(Nb)ICs/RHEAs for which such data are available, and cannot include RCCAs or RHEAs, for example, like those included in the review in [19], owing to lack of data for the latter metallic UHTMs.

## 2. Complex Concentrated Bcc Solid Solution

The bcc Nb_ss_ in RM(Nb)ICs and RM(Nb)ICs/RCCAs with nominal Si concentration 18 at.% and alloying addition of Al, B, Cr, Ge, Hf, Mo, Nb, Sn, Ta, Ti or W can be (i) “conventional” Nb_ss_, (ii) CC/HE Nb_ss_, or (iii) Nb_ss_ with no Si and (iv) not stable after heat treatment. These types of bcc solid solution are shown schematically in Figure 1 where the colours for (i) to (iv) are dark blue, red, light purple and light blue, respectively. Note that Figure 1 has data for bcc Nb_ss_ in AC and HT alloys. “Conventional” Nb_ss_ can be Ti rich only in AC alloys, meaning Ti rich Nb_ss_ is not observed in HT alloys. In B containing alloy the Ti rich solid solution is usually CC/HE Nb_ss_.

For presentation purposes, in Figure 1 the numbers 15, 10 and 5 have been assigned, respectively, to “conventional” Nb_ss_, CC/HE Nb_ss_ and not stable Nb_ss_. The nominal compositions of the alloys are shown in the Appendix A. For most of the alloys in Figure 1 the CC/HE Nb_ss_ that was formed in the AC alloy was not stable after heat treatment.

The data in Figure 1 show (a) that CC Nb_ss_ was stable after heat treatment only in alloys where Mo and W simultaneously were in synergy with Hf and with the simultaneous addition of Ge and Sn in the alloy (compare the alloys JZ3+, JZ4, JZ5 and the OHS1), whereas (b) when Mo was substituted with Ta a higher concertation of Sn was required to stabilise the CC Nb_ss_ in the heat treated alloy (compare the alloys JZ3+ and JZ3). The (a) is also supported by the data for the alloy JN2, which in the AC condition had “conventional” Nb_ss_ plus two CC solid solutions and only “conventional” Nb_ss_ in the HT condition [51]. Furthermore, (c) in the alloy JZ3+ the CC Nb_ss_ was formed in the AC and HT conditions, whereas (d) in the alloys JZ4 and JZ5 Nb_ss_ was not formed in the AC condition and the CC Nb_ss_ with no Si formed after heat treatment, while (e) the opposite was the case in the alloy OHS1, where CC Nb_ss_ was formed in the AC condition and the Nb_ss_ was not stable after heat treatment. In the B containing TT4, TT5, TT7 and TT8 alloys and the Ta containing alloy KZ6 “conventional” and CC/HE Nb_ss_ formed in the AC condition and only “conventional” Nb_ss_ after heat treatment, but in the Sn containing alloys EZ8, JG6 and OHS1 the CC Nb_ss_ was formed in the AC condition and the Nb_ss_ was not stable after heat treatment. In the alloys EZ5 and TT6, “conventional” Nb_ss_ formed in the AC condition and the Nb_ss_ was not stable after heat treatment. Note that both alloys contain Sn, whereas B was present only in the alloy TT6.

In other words, considering the three elements B, Ge and Sn, which in synergy with Al, Cr, Hf and Ti, are key for improving the oxidation resistance and obtaining a balance of properties in RM(Nb)ICs, RM(Nb)ICs/RCCAs and RM(Nb)ICs/RHEAs [3,9,10,14,17,18,33,34,35,36,42,52,53,54] and (I would suggest) in RCCAs and RHEAs, it is advised that alloying with B plus Hf or Mo or Ta is unlikely to stabilise CC/HE Nb_ss_ compared with the simultaneous addition of Ge and Sn with Hf, Mo and W in the aforementioned metallic UHTMs.

**Figure 2:**
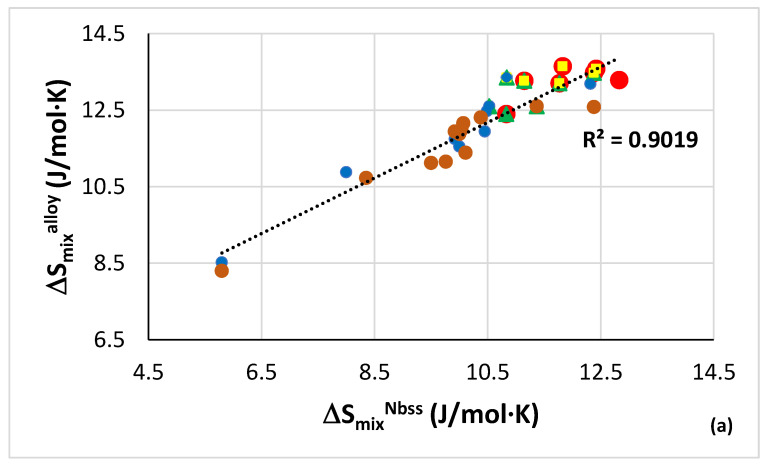

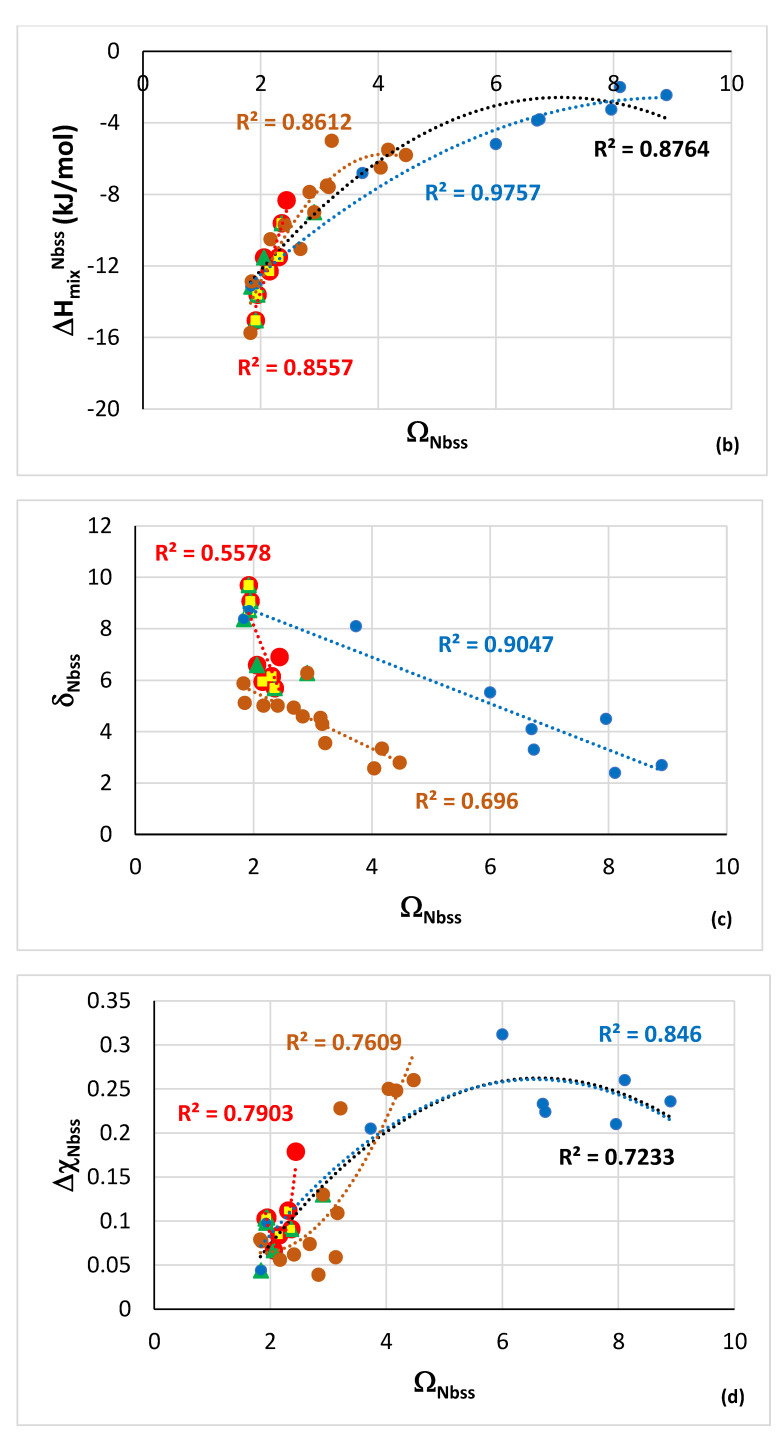
(**a**) Alloy entropy of mixing versus solid solution entropy of mixing, and (**b**–**d**) relationships of the solid solution parameter Ω with the solid solution (**b**) enthalpy of mixing, (**c**) parameter δ and (**d**) parameter Δχ. Red data CC/HE Nbss alloys JN2-AC, TT4-AC, TT7-AC, EZ8-AC, TT6-AC, ZF9-AC, TT5-HT, blue and brown data “conventional” Nbss, blue data AC and HT alloys JN3 and JN4, and HT alloys YG8, YG10, TT4, TT7, brown data AC alloys YG8, YG10, AC and HT alloys YG11, KZ5, JN1 and ZF6, and HT alloys KZ6, JG3 and TT8. Green triangles for B containing alloys, yellow squares for RCCAs. For nominal alloy compositions and references see the Appendix A. In (**a**) for all data R^2^ = 0.9019, blue and brown data linear fit with R^2^ = 0.9127, brown data linear fit with R^2^ = 0.8854, in (**b**) R^2^ = 0.8557 is for linear fit and R^2^ = 0.9757, R^2^ = 0.8612 and R^2^ = 0.8764 are for parabolic fit, the latter value is for all the data, in (**c**) all the R^2^ values are for linear fit of data, in (**d**) all the R^2^ values are for parabolic fit, and R^2^ = 0.7233 is for all the data. HE Nb_ss_ in TT4-AC.

The relationship between the entropies of mixing of alloys and their bcc solid solutions is shown in Figure 2a. Data for solid solutions and alloys can be found, respectively, in the Table 1 in [28] and the Table 1 in [26] and the nominal alloy compositions are given in the Appendix A. In Figure 2a the linear fit of all the data is good (R^2^ = 0.9019) and shows that the CC/HE Nb_ss_ has high entropy of mixing (see below), and is formed in RM(Nb)ICs, and RM(Nb)ICs/RCCAs or RM(Nb)IC/RHEAs that also have high entropy of mixing (12.4 < ΔS_mix_^alloy^ < 13.65 Jmol^−1^K^−1^). Relationships of the solid solution parameter Ω with the solid solution enthalpy of mixing, and the parameters δ and Δχ are shown in Figure 2b–d. The CC/HE Nb_ss_ has low Ω (<2.4), high |ΔH_mix_|, high and low δ and Δχ (Pauling electronegativity) parameters (>5.7 and <0.18, respectively) and low VEC (figure not shown). In the plots of Ω_ss_ with δ_ss_, Δχ_ss_ and VEC_ss_ (figure not shown) only δ_ss_ can show the effect of specific alloying additions. Indeed, in Figure 2c the blue data are for the alloying additions Al, B, Cr, Hf, Mo, Nb, Si, Sn, Ti and W, the brown data for Al, B, Cr, Ge, Hf, Mo, Nb, Si, Ta, Ti, W and the red data for Al, B, Cr, Ge, Hf, Mo, Nb, Si, Sn, Ta, Ti and W. Note that the blue and brown lines in Figure 2c are essentially parallel, and that the red line is for alloys with 24 at.% Ti and 18 at.% Si (nominal). In other words, (i) the addition of Ta and the replacement of Sn with Ge reduces both the δ_ss_ and Ω_ss_ parameters (shift from blue to brown line), whereas the simultaneous addition of the said elements “bridges the gap” with further decrease in Ω_ss_ and formation of CC/HE Nb_ss_ (red data) and (ii) the parameters δ_ss_ and Ω_ss_ are key in the alloy design stage for designing alloys with “conventional” and CC/HE Nb_ss_.

How do the values of the parameters for CC/HE Nb_ss_ of the alloys in Figure 2 compare with those of single-phase bcc solid solution HEAs? Whereas there are similarities for the entropy of mixing (10.8 < ΔS_mix_^CC/HE Nbss^ < 12.8 Jmol^−1^K^−1^, compared, for example, with 11.47, 11.53 and 13.38 Jmol^−1^K^−1^, respectively, for the HE_ss_ Hf_21_Mo_20_Nb_21_Ti_17_Zr_21_, WNbMoTa and WNbMoTaV) and VEC_CC/HE Nbss_ (4.44 < VEC_CC/HE Nbss_ < 4.74, compared, for example, with 4.4, 4.6, 4.7, 5.5 and 5.4, respectively, for the high entropy solid solution (HE_ss_) HfNbTaTiZr, HfMoTaTiZr, HfMoNbTaTiZr, WNbMoTa and WNbMoTaV) there are significant differences for the other parameters. Indeed, the δ_CC/HE Nbss_ values are higher (5.7 < δ_CC/HE Nbss_ < 9.7, compared, for example, with 2.31, 3.15, 5.51 and 6.3, respectively, for the HE_ss_ WNbMoTa, WNbMoTaV, HfNbTaTiZr and HfMoNbTaTiZr), the Ω_CC/HE Nbss_ values are lower (1.9 < Ω_CC/HE Nbss_ < 2.44, compared, for example, with 12.37, 17.8, 24.9 and 43.3, respectively, for the HE_ss_ HfNbTaTiZr, HfMoTaTiZr, HfMoNbTaTi and HfMoNbTaTiZr), the enthalpy of mixing is more negative (−15.04 < ΔH_mix_^CC/HE Nbss^ < −8.32 KJ mol^−1^, compared, for example, with −0.9, −1.9, −4.64 and −6.5 KJ mol^−1^, respectively, for the HE_ss_ HfMoNbTaTiZr, HfMoTaTiZr, WNbMoTaV and WNbMoTa) and Δχ_CC/HE Nbss_ values are smaller (0.067 < Δχ_CC/HE Nbss_ < 0.179, compared, for example, with 0.34 and 0.36, respectively, for the HE_ss_ WNbMoTaV and WNbMoTa).

The higher values of δ_CC/HE Nbss_ are attributed to the alloying with B, the more negative ΔH_mix_^CC/HE Nbss^, and the low Ω_CC/HE Nbss_ and Δχ_CC/HE Nbss_ values are attributed to the alloying with B, Ge or Sn. The aforementioned alloying elements have not been used in studies of single-phase bcc solid solution HEAs.

**Figure 3:**
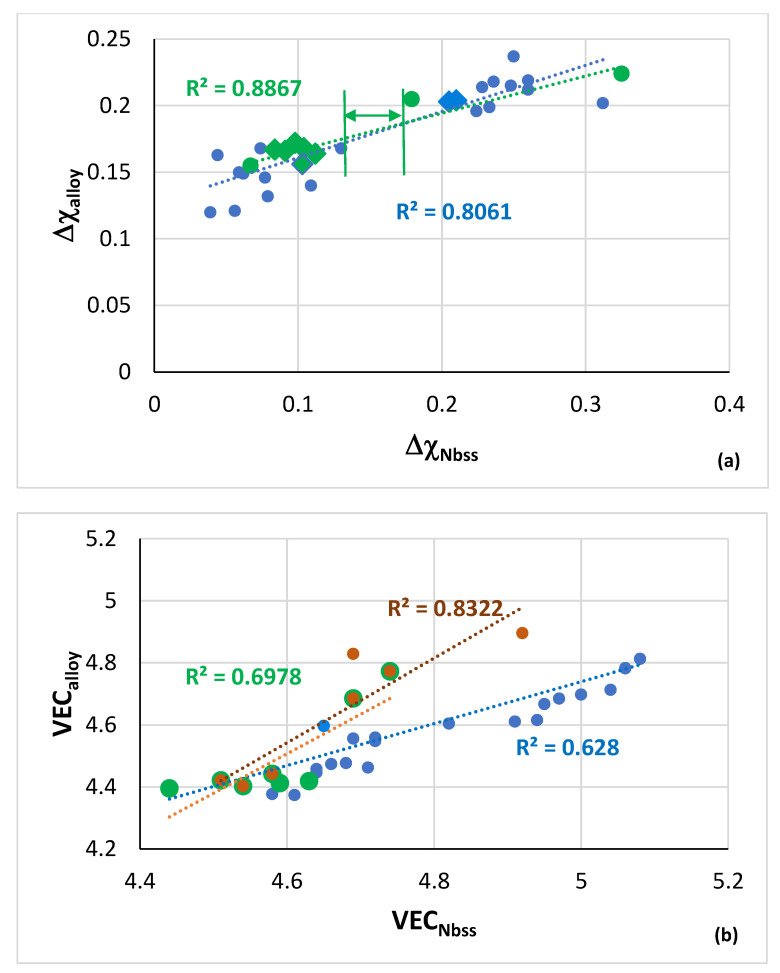
(**a**) Δχ_alloy_ versus Δχ_Nbss_ and (**b**) VEC_alloy_ versus VEC_Nbss_. The data are for the same alloys as in Figure 2. In (**a**) green data points R^2^ = 0.8867, all data points R^2^ = 0.8061. In (**b**) the brown data points (R^2^ = 0.8322) are for the alloys JN2-AC, YG8-HT, YG11-HT, TT7-AC, EZ8-AC, TT5-HT, the green data points (R^2^ = 0.6978) are for the alloys JN2-AC, TT4-AC, TT7-AC, EZ8-AC, TT6-AC, ZF9-AC, TT5-AC, and the blue data points (R^2^ = 0.628) are for the alloys JN2-HT, JN3, JN4, YG8-AC, YG10, YG11-AC, KZ5, KZ6-HT, JN1, TT4-HT, TT7-HT, ZF6, JG3-HT, TT8-HT (see the Appendix A for nominal alloy compositions and references). In (**a**) the diamonds indicate RM(Nb)IC/RCCA. Diamonds not shown in (**b**) for clarity of presenting the different groups. HE Nb_ss_ in TT4-AC.

Relationships between the parameters Δχ and VEC of alloys and Nb_ss_ are shown in Figure 3, where the CC/HE Nb_ss_ is indicated with the green data points. Note that this type of solid solution was mostly observed in AC alloys (Figure 1). The data in Figure 3 are for the same alloys as in Figure 2. In Figure 3a the R^2^ = 0.8061 is for the linear fit of all the data and R^2^ = 0.8867 is for the data of the CC/HE Nb_ss_. Notice (i) the gap (green double arrow) in Δχ_Nbss_ values, in agreement with [28], which means that the CC/HE Nb_ss_ follows the same rules as the “conventional” Nb_ss_ [10], and (ii) that CC/HE Nb_ss_ is found on either side of this gap. In Figure 3b all the data have R^2^ = 0.628, the brown data points give R^2^ = 0.8322, and the green data points give R^2^ = 0.6978.

Even though the same alloying additions were in the alloys and their solid solutions represented by the green and blue data points in Figure 3, the alloys and their solid solutions indicated with the brown data points did not contain Ge and their Ti content was not fixed at 24 at.% nominal, as is the case for the alloys represented with the green data points. Instead, they were either Ti free (alloy YG8) or their Ti concentration was lower (alloys YG10, YG11). In other words, the parameter VEC (Figure 3b) shows that not only the alloying additions but also their concentrations in an alloy are key regarding the formation or not of CC/HE Nb_ss_. Furthermore, only with the parameter VEC we can differentiate the data for CC/HE Nb_ss_ and “conventional” Nb_ss_, as indicated with the brown and green lines compared with the blue line in Figure 3b. Thus, the co-existence of CC/HE Nb_ss_ with “conventional” Nb_ss_ in most alloys [14] is supported by the data in Figure 2 and Figure 3. Additionally, Figure 3 confirms (iii) that the relationships between the alloy and solid solution parameters Δχ and VEC, which are fundamental relationships in NICE [10], apply also for CC/HE Nb_ss_ and (iv) that the parameters VEC_alloy_ and VEC_ss_ are key in the alloy design stage for designing alloys with “conventional” and CC/HE Nb_ss_. To summarise, the design of alloys with “conventional” and CC/HE Nb_ss_ must make use of the relationships of the parameter Δχ_alloy_ with the concentrations of solute additions in NICE [10] and the relationships between the parameters VEC_alloy_ and VEC_ss_ and δ_ss_ and Ω_ss_.

**Figure 4:**
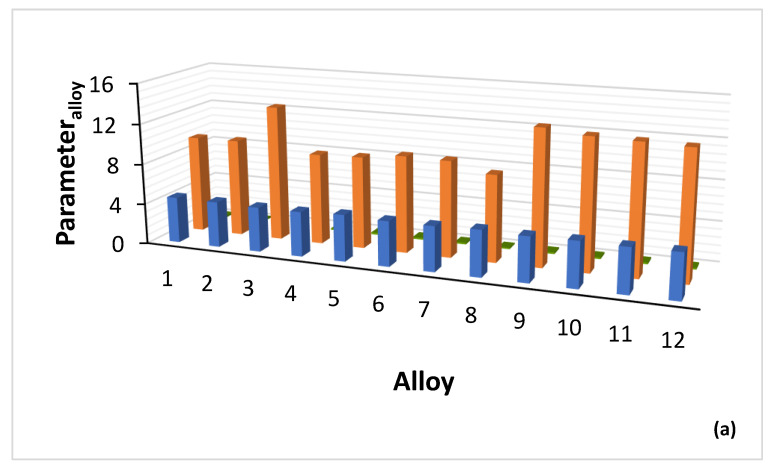

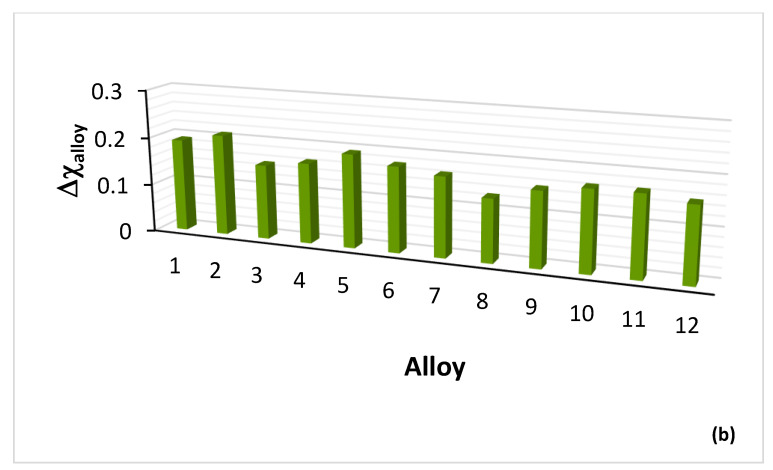
(**a**) Parameters VEC, δ and Δχ of alloys in which CC/HE Nb_ss_ was observed and (**b**) details of Δχ_alloy._ Colours: blue VEC, brown δ, green Δχ. Alloys 1 to 12 contain Al, Cr, Nb, Si, Ti plus in (1) Ge, Hf, Sn, Ta, W, in (2) Ge, Hf, Mo, Sn, W, in (3) B, Ta, in (4) Ge, Hf, in (5) Ge, Hf, Sn, Ta, W, in (6) Hf, Mo, Sn, in (7) Hf, Sn, in (8) Ta, in (9) B, in (10), B, Mo, in (11) B, Hf, in (12) B, Ta. 1 = JZ3+-AC, 2 = JZ5-HT, 3 = TT5-AC, 4 = ZF9-AC, 5 = JZ3-AC, 6 = JG6-AC, 7 = EZ8-AC, 8 = KZ6-AC, 9 = TT4-AC, 10 = TT8-AC, 11 = TT7-AC, 12 = TT5-HT. For nominal alloy compositions and references see Appendix A. RM(Nb)ICs (5, 8–10) and RM(Nb)ICs/RCCAs (1–4,6,7,11,12).

**Figure 5:**
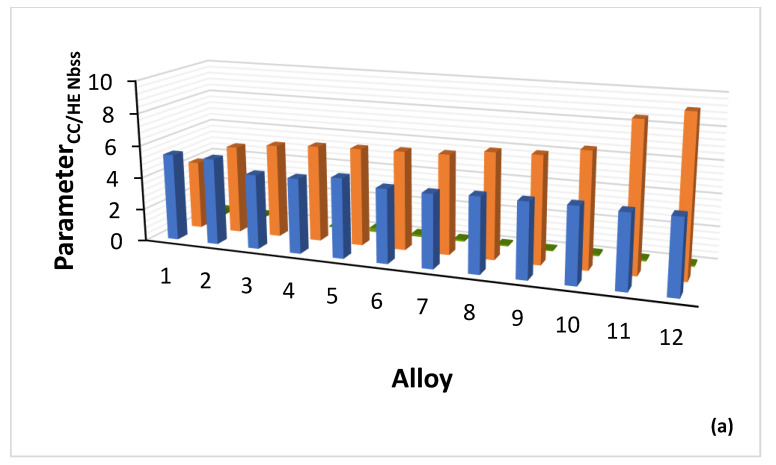

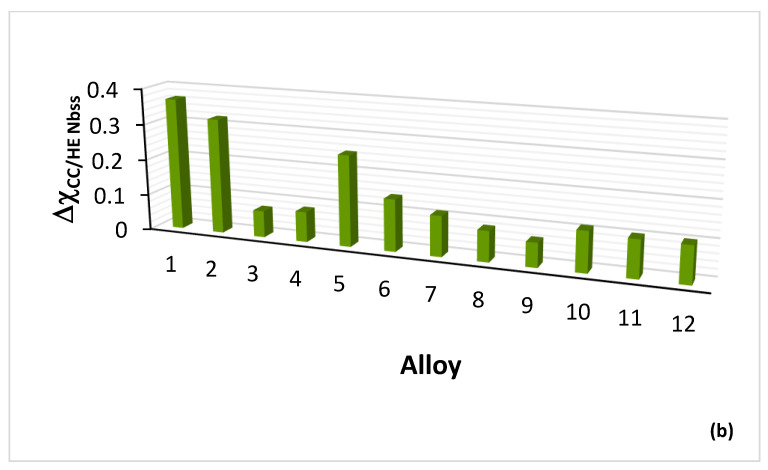
(**a**) Parameters VEC, δ and Δχ of CC/HE Nb_ss_ in alloys where this type of solid solution was observed and (**b**) details of Δχ_CC/HE Nbss._ Colours: blue VEC, brown δ, green Δχ. Alloys 1 to 12 the same as in Figure 4. HE Nb_ss_ in TT4-AC. For nominal alloy compositions and references see Appendix A. RM(Nb)ICs (5, 8–10) and RM(Nb)ICs/RCCAs (1–4,6,7,11,12).

**Figure 6:**
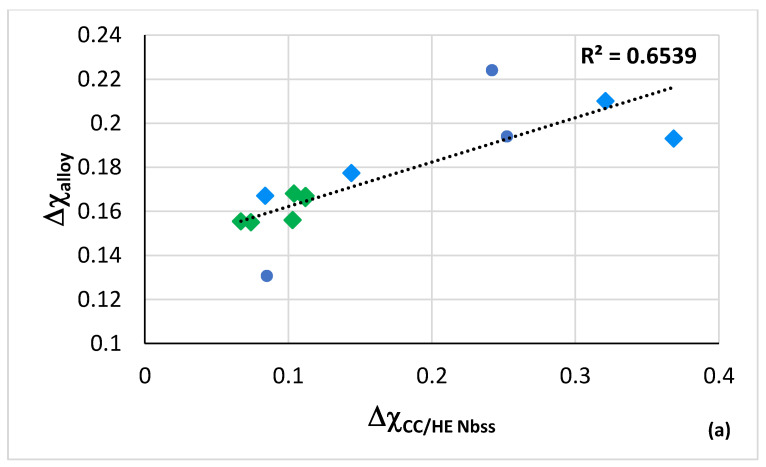

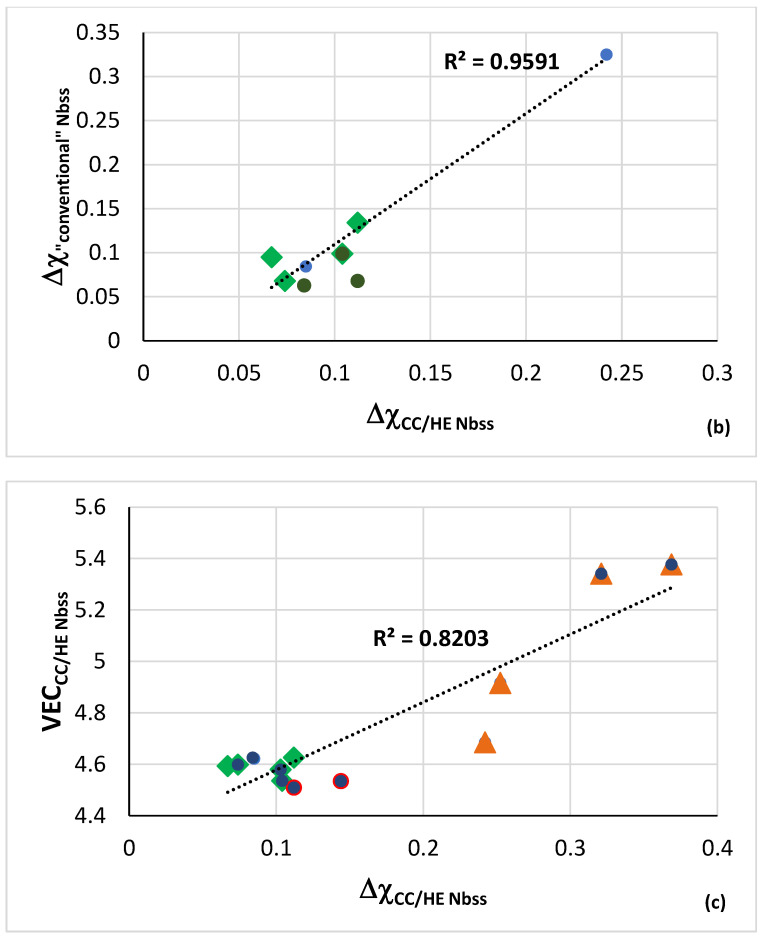
(**a**) Δχ_alloy_ versus Δχ_CC/HE Nbss_, where green colour indicates B containing alloys and diamonds are for RM(Nb)ICs/RCCAs. (**b**) Δχ_”conventional” Nbss_ versus Δχ_CC/HE Nbss_, where green diamonds are solid solutions in B containing alloys and green circles for solid solutions in RM(Nb)ICs/RCCAs, (**c**) VEC_CC/HE Nbss_ versus Δχ_CC/HE Nbss_, where green diamonds are for solid solutions in B containing alloys, brown triangles are for solid solutions in alloys with simultaneous addition of Ge and Sn, red circles are for solid solutions with Sn, and blue circles are for solid solutions in RM(Nb)ICs/RCCAs. In each part the R^2^ value is for the linear fit of all the data. (**a**,**c**) data for the AC alloys EZ8, JG6, JZ3, JZ3+, KZ6, TT4, TT5, TT7, TT8, ZF9 and the HT alloys JZ5 and TT5, (**b**) data for the AC alloys KZ6, TT4, TT5, TT7, TT8, ZF9. HE Nb_ss_ in TT4-AC. See the Appendix A for nominal alloy compositions and references.

**Figure 7:**
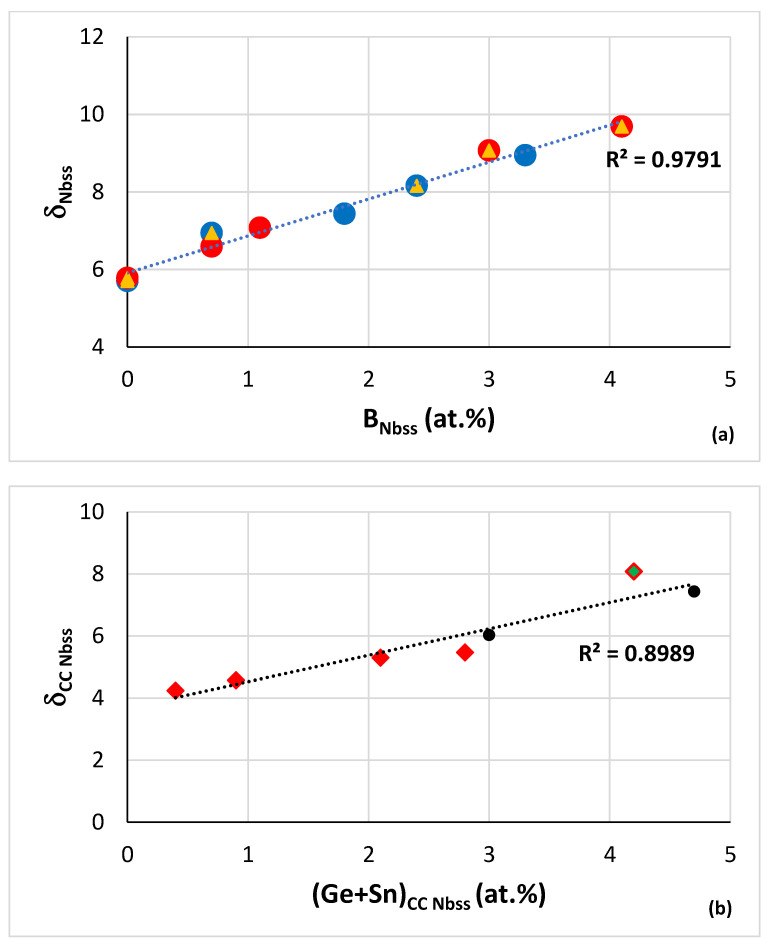
(**a**) Parameter δ of solid solution versus its B concentration. Red data points for CC/HE Nb_ss_, blue data points for “conventional” Nb_ss_. Yellow triangles indicate solid solution was formed in RM(Nb)IC/RCCA. All data R^2^ = 0.9791, data for CC/HE Nb_ss_ has R^2^ = 0.9884 and data for “conventional” Nb_ss_ has R^2^ = 0.9656. Data are as follows: “conventional Nb_ss_ in AC alloys TT4, TT5, TT6, TT7, TT8, CC/HE Nb_ss_ for the AC alloys TT4, TT5, TT7, TT8 and the HT alloy TT5. HE Nb_ss_ in TT4-AC. (**b**) Parameter δ versus (Ge+Sn) content of CC Nb_ss_. Data for the AC alloys JZ3, JZ3+ and OHS1 and the HT alloys JZ3+, JZ4 and JZ5. All data R^2^ = 0.8989. Diamonds for solid solutions in RM(Nb)ICs/RCCAs. Green colour for the solid solution in the alloy OHS1. For nominal alloy compositions and references see Appendix A.

The parameters VEC, δ and Δχ of alloys in which CC/HE Nb_ss_ was observed are shown in Figure 4, and the parameters VEC, δ and Δχ of the CC/HE Nb_ss_ in the same alloys are shown in Figure 5. Figure 4a shows significantly higher values of δ_alloy_ for B containing alloys (range 12.57 to 13.35, compared with 8.55 to 9.66 for B free alloys) and essentially similar VEC_alloy_ values (4.403 to 4.584). Figure 4b shows small range of Δχ_alloy_ values (0.131 to 0.21) (also see Table 1 in [26]), and wider range and higher values of VEC_CC/HE Nbss_ (4.51 to 5.38, Figure 5a). Significantly wider range of Δχ_CC/HE Nbss_ values (0.067 to 0.369) is shown in Figure 5b with strikingly lower values for B containing CC Nb_ss_ (Figure 6a), noticeably higher values of δ_CC/HE Nbss_ for RM(Nb)ICs/RCCAs where B was simultaneously present with Hf (alloy 11) or Ta (alloy 12) (Figure 5a) and overall markedly lower values of δ_CC/HE Nbss_ (4.239 to 9.69) compared with δ_alloy_ (also see Table 1 in [28]). The parameter Δχ_CC/HE Nbss_ increases with increasing Δχ_alloy_ (Figure 6a), Δχ_”conventional” Nbss_ (Figure 6b) and VEC_CC/HE Nbss_ (Figure 6c). Remarkably, there is a strong correlation between the δ parameters of “conventional” and CC/HE Nb_ss_ with the B concentration of the solid solution, as shown in Figure 7a.

The co-existence of CC/HE Nb_ss_ with “conventional” Nb_ss_ in most alloys [14] is further supported by the data in Figure 6 that also confirm that the relationships between the alloy and solid solution parameters Δχ and VEC, which are fundamental relationships in NICE [10], apply also for CC/HE Nb_ss_.

Boron, Ge and Sn are key elements for obtaining a balance of properties in metallic UHTMs but their roles regarding the stability of CC/HE Nb_ss_ differ, see above. The CC/HE Nb_ss_ was stable after heat treatment in alloys with simultaneous addition of Mo, W with Hf, Ge and Sn (Figure 1). Figure 7 shows relationships of the solid solution parameter δ versus the B or Ge+Sn concentration in the solid solution. In both cases the parameter δ increases with increasing B or Ge+Sn concentration in the solid solution.

Note that in Figure 7a, the data are for “conventional” and CC/HE Nb_ss_, whereas in Figure 7b the data are only for CC/HE Nb_ss_. The co-existence of CC/HE Nb_ss_ with “conventional” Nb_ss_ in boron containing alloys is further supported by the data in Figure 7a. Lowest B concentration in the solid solution and thus lowest δ parameter was found when B was simultaneously present with Sn or Ta in the alloy [35]. Correlations of boron concentration in Nb_ss_ with the parameters VEC and Δχ are not strong (figures not shown).

**Figure 8:**
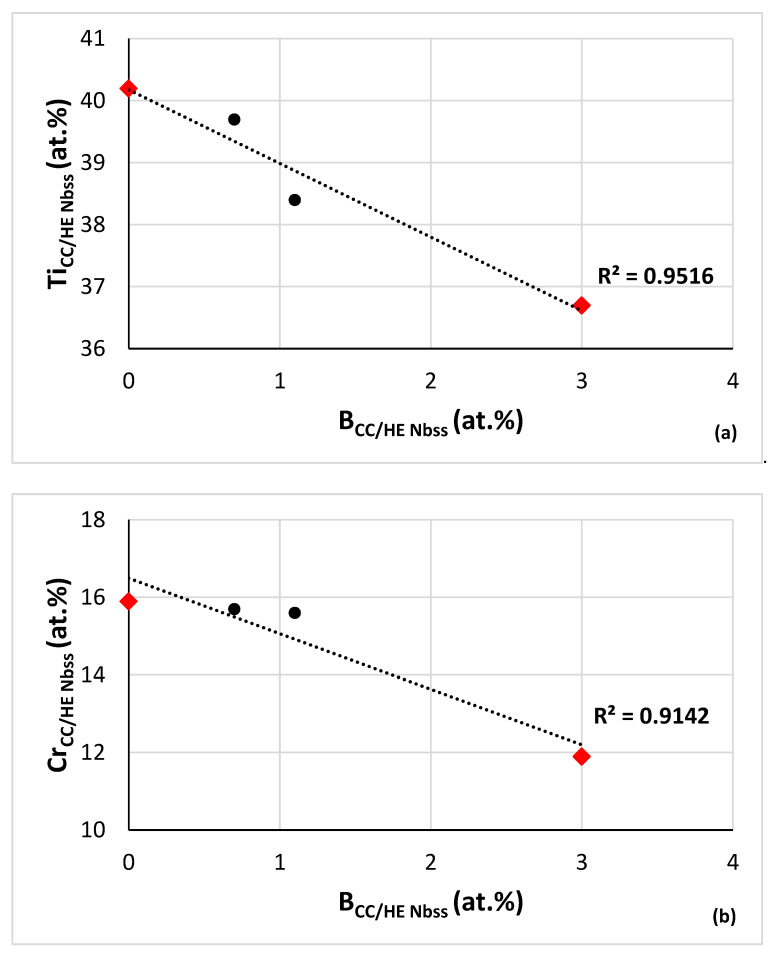

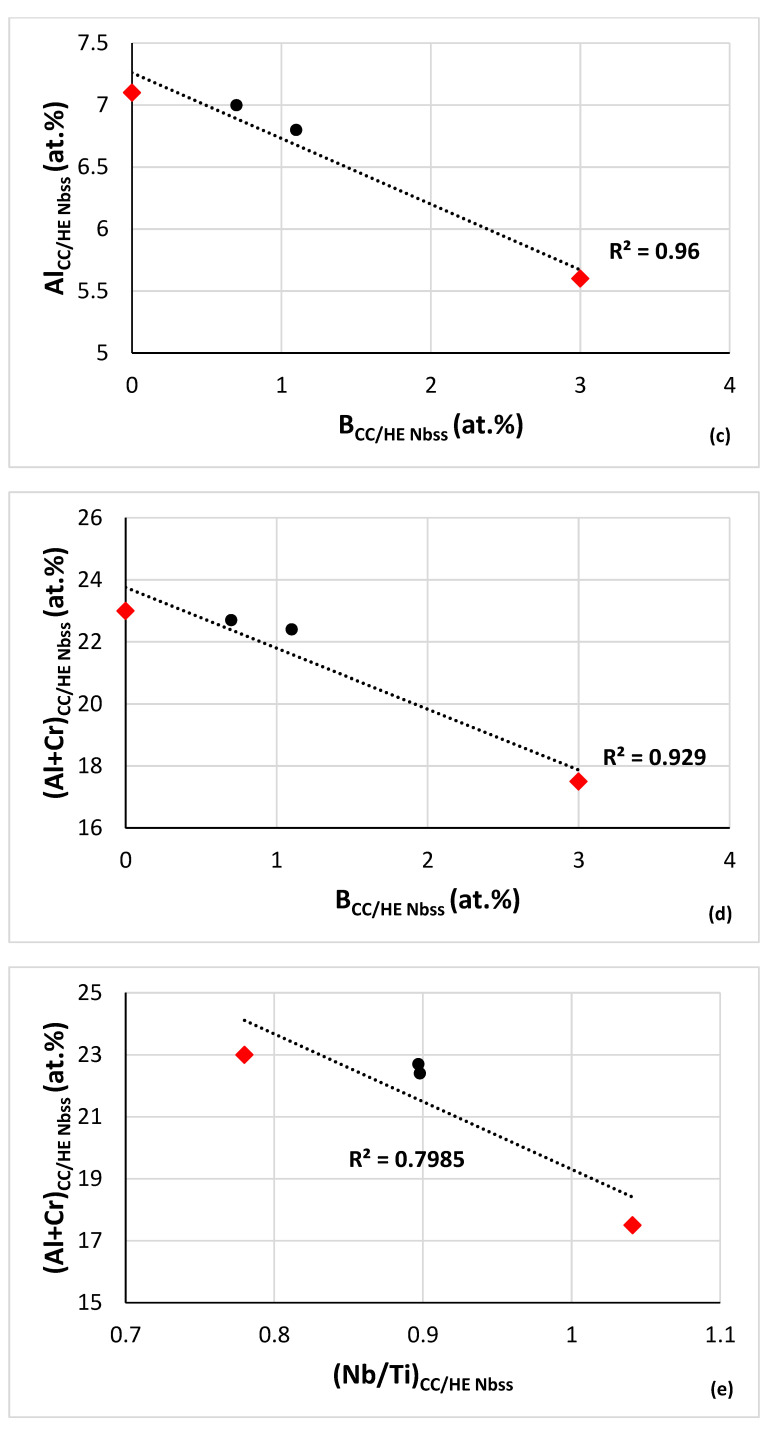

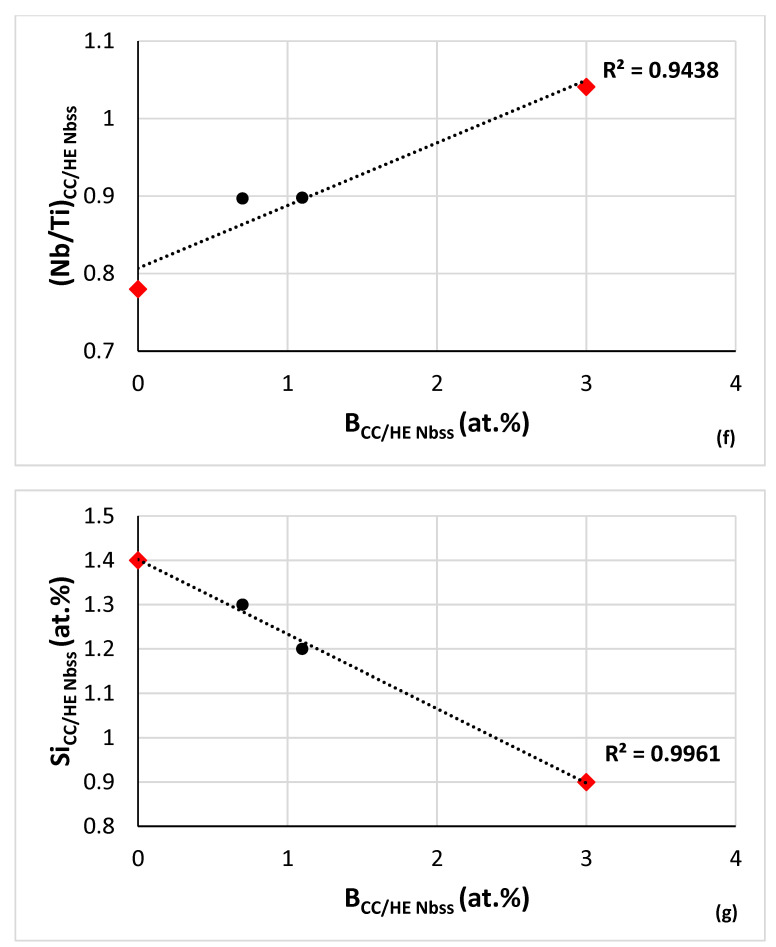
(**a**–**g**) data for the as cast B containing alloys TT4, TT5, TT7, TT8. Diamonds for RM(Nb)ICs/RCCAs. Concentration of B versus (**a**) Ti, (**b**) Cr, (**c**) Al, (**d**) Al+Cr and (**g**) Si in CC/HE Nb_ss_. (**e**) Al+Cr concentration versus Nb/Ti ratio and (**f**) Nb/Ti ratio versus B concentration in CC/HE Nb_ss_. R^2^ values are for the linear fit of all data in each part. Parabolic fit of data in (**e**) gives R^2^ = 0.9981 with maximum at Nb/Ti = 0.82 and (Al+Cr) = 23.22 at.%. HE Nb_ss_ in TT4-AC. For nominal alloy compositions and references see Appendix A.

**Figure 9:**
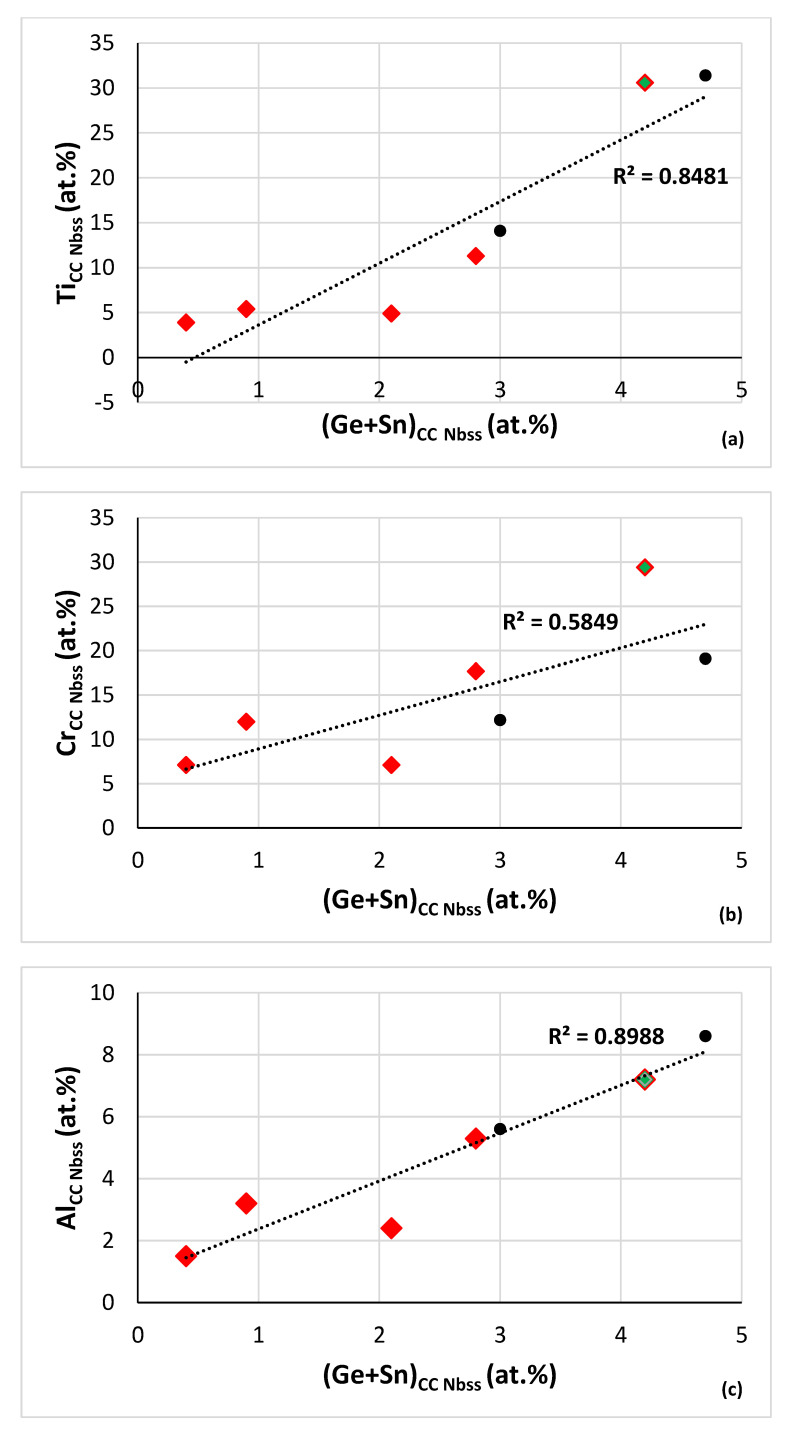

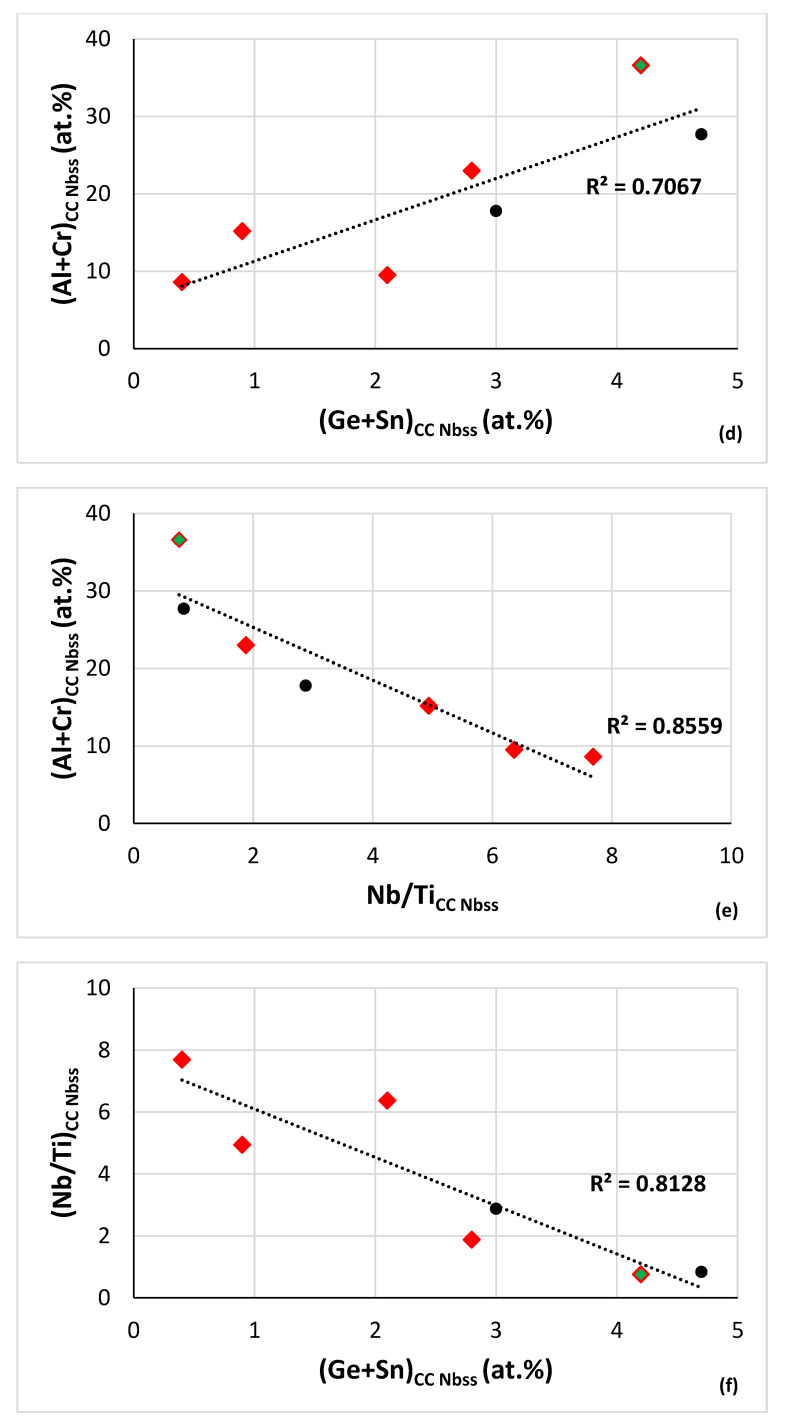

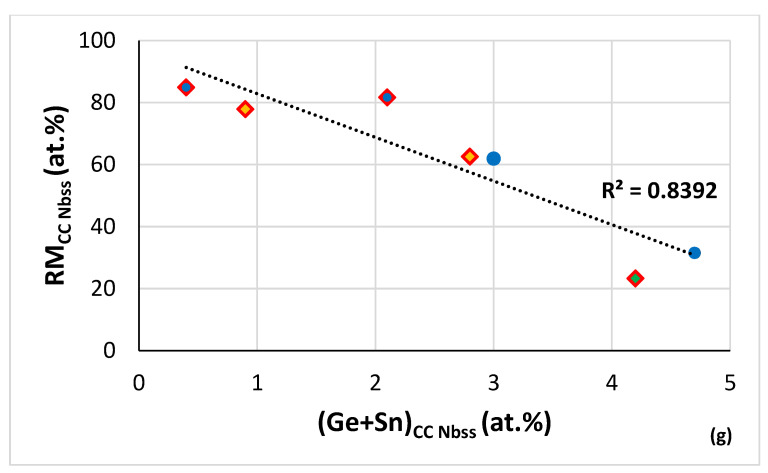
(**a**–**g**) data for the AC alloys JZ3, JZ3+, OHS1 and the HT alloys JZ3+, JZ4, JZ5 with simultaneous addition of Ge+Sn. Concentration of (Ge+Sn) versus (**a**) Ti, (**b**) Cr, (**c**) Al, (**d**) Al+Cr in CC Nb_ss_. (**e**) Al+Cr concentration versus Nb/Ti ratio, (**f**) Nb/Ti ratio versus (Ge+Sn) concentration and (**g**) total RM (=Nb + Mo + Ta + W) concentration versus (Ge+Sn) concentration in CC Nb_ss_. R^2^ values are for the linear fit of all data in each part. Solid solution in RM(Nb)IC/RCCA alloy indicated with diamond and the green colour is for the alloy OHS1. In (**g**) blue colour for alloys where RM = Nb + Ta + W, yellow for RM = Nb + Mo + W and green for RM = Nb, i.e., for the alloy OHS1. See Appendix A for nominal alloy compositions and references.

Relationships between B or Ge+Sn concentration and other solute additions in CC/HE Nb_ss_ in RM(Nb)ICs and RM(Nb)ICs/RCCAs are shown in Figure 8 and Figure 9. Figure 8a–d shows relationships of B concentration with “reactive” solutes in CC/HE Nb_ss_ in boron containing RM(Nb)ICs and RM(Nb)ICs/RCCAs and Figure 8e,f shows correlations with the Nb/Ti ratio in the CC/HE Nb_ss_. The correlation between the B and Si concentrations in the solid solution is shown in Figure 8g. In Figure 8 the solid solution in RM(Nb)ICs/RCCAs is indicated with diamonds. The same correlations for “conventional” Nb_ss_ are not strong (figures not shown).

The Al, Cr, Ti, Al+Cr and Si concentrations in the CC/HE Nb_ss_ decrease as its boron concentration increases. The Nb/Ti ratio of the CC/HE Nb_ss_ increases with its boron concentration and decreases with its Al+Cr content. The parabolic fit of data in Figure 8e give R^2^ = 0.9981 with maximum for Nb/Ti = 0.82 and (Al+Cr) = 23.22 at.%. For Nb/Ti = 0.82 Figure 8f gives B = 0.16 at.%. Using this B concentration, from Figure 8a we obtain Ti = 39.98 at.%, from Figure 8b Cr = 16.26 at.%, from Figure 8c Al = 7.18 at.%, from Figure 8d Al+Cr = 23.44 at.%, from Figure 8g Si = 1.37 at.%. Finally, for Ti = 39.98 at.% and the ratio Nb/Ti = 0.82 we obtain Nb = 32.78 at.%, in other words we calculate the chemical composition of CC Nb_ss_ as 32.78Nb-39.98Ti-16.26Cr-7.18Al-1.37Si-0.16B or 32.8Nb-40Ti-16.3Cr-7.2Al-1.4Si-0.2B.

Whereas the Al, Cr, Ti and Al+Cr concentrations in the CC/HE Nb_ss_ in B containing alloys decrease as the B concentration in the solid solution increases (Figure 8), the opposite is the case when the concentrations of the same solute additions are plotted versus the Ge+Sn concentration of the CC Nb_ss_ in B free alloys (Figure 9). Note that there is no correlation between the Si and Ge+Sn concentrations in CC Nb_ss_. Similarly with the B containing alloys, the Nb/Ti ratio of the CC Nb_ss_ increases with decreasing Al+Cr content (Figure 8e and Figure 9e), but unlike the B containing Nb_ss_, the Nb/Ti ratio decreases with increasing Ge+Sn concentration (Figure 8f and Figure 9f). Furthermore, there is a good correlation between the total RM concentration in CC Nb_ss_ and its Ge+Sn content that shows the former decreasing as the latter content increases (RM = Nb + Mo + Ta + W). Note that also there are good correlations between the W and Ti content, the Ti concentration with the W/RM ratio and the Al+Cr sum with the Sn/Ge ratio of the Nb_ss_ of B free RM(Nb)ICs and RM(Nb)ICs/RCCAs with Ge, Sn, and RM additions (see Figure 12 in [33] and Figure 12 in [34]).

In B containing RM(Nb)ICs and RM(Nb)ICs/RCCAs, the parameter δ_Nbss_ increases with B_Nbss_ (Figure 7a) and the hardness of the solid solution decreases with increasing δ_Nbss_ (see the descending part (green data) of the HV_ss_ versus δ_ss_ data in Figure 7 in [3]). From the two linear relationships the dependence of HV_ss_ on B_Nbss_ can be derived. The hardness of Nb_ss_ in B free or B containing alloys is discussed in the next section. Note that the alloying with B has the opposite effect on the hardness of tetragonal Nb_5_Si_3_ compared with the effect of Ge or Sn, meaning the hardness increases upon alloying with B (see Figure 14 in [9]).

The ductile behaviour and yield strength of bcc Nb-rich solid solution alloys with Al, Cr and Ti additions (i.e., (Nb,Ti,Cr,Al)_ss_) has been studied for different Nb/Ti ratios and Al+Cr sums [55]. At low Nb/Ti ratios, brittle behaviour was observed at higher Al+Cr content compared with high Nb/Ti ratios. For example, for Nb/Ti ≈ 0.8 brittle behaviour was observed for Al+Cr higher than about 22 at.%, and for Nb/Ti ≈ 1 or 2 ductile behaviour was observed for Al+Cr less than about 20 at.% and 18 at.%, respectively,. The room temperature yield strength decreased with decreasing Nb/Ti ratio. For example, for Nb/Ti ≈ 1 and Al+Cr ≈ 20 at.% the yield strength was about 980 MPa, whereas for Nb/Ti ≈ 0.8 it was about 825 MPa for Al+Cr ≈ 22 at.%. For Nb/Ti ≈ 1 increasing the Al content gave strengthening at room temperature and weakening at high temperatures, the Cr addition gave significant strengthening at all temperatures, approximately doubling the strength at 1200 °C. Reduced Ti concentration improved the high temperature strength. Note that for B containing RM(Nb)ICs and RM(Nb)ICs/RCCAs the Nb/Ti ratio of the solid solution increases with increasing B content (Figure 8f). It is suggested that it would be possible to “ductilize” the “conventional” or CC/HE Nb_ss_ with B addition and “fine tuning” of the Nb/Ti ratio, and the Al+Cr sum of the Nb_ss_ in multiphase RM(Nb)ICs, RM(Nb)ICs/RCCAs and RM(Nb)ICs/RHEAs (Figure 8).

Unlike the B containing RM(Nb)ICs and RM(Nb)ICs/RCCAs, currently there are no hardness data for the solid solutions in B free alloys with Ge, Sn and RM additions. Like the B-containing alloys, the latter alloys (i) exhibit exceptional oxidation resistance at pest and high temperatures with no scale spallation [33,34,35,36] and (ii) are expected to have good creep properties [14,34]. A material system suitable for high pressure turbine comprising a RCCA substrate of the Nb-Al-Cr-Ge-Hf-Mo-Si-Sn-Ti-W alloy system and a HEA bond coat of the Nb-Al-Hf-Si-Ti alloy system has been proposed using NICE [14,34].

## 3. Contamination of the Bcc Solid Solution with Oxygen

### 3.1. Contamination of Nb with Interstitials

The contamination of Nb with carbon, hydrogen, nitrogen and oxygen has been reported in the literature. Pionke and Davis found out that in the temperature range 200 to 600 °C, both carbon and nitrogen had very limited solubility in Nb (<0.1 at.% (0.014 wt.%)), oxygen had slightly more (<0.6 at.%, (0.1 wt.%)) while hydrogen had very large solubility, about 10 at.% (0.1 wt.%). Unlike the other interstitial elements, the solubility of hydrogen in Nb decreased with increasing temperature. The equilibrium concentration of hydrogen was affected by pressure [56].

The use of reactive alloying elements (Hf, Ti, and Zr) in Nb tends to lower the oxygen solubility. The addition of Zr is of particular interest because Zr is an effective strengthener of Nb. Zirconium additions to Nb have the effect of lowering the apparent oxygen solubility limit but increasing the Nb solubility limit. This increase is roughly a factor of 4 for a given temperature and pressure [57].

There are conflicting reports about the solution hardening of Nb with oxygen and nitrogen. For example, Harris [58] reported that oxygen was three times more effective in solution hardening than nitrogen or carbon, whereas Seigle [59] found the latter two elements to be twice as effective as oxygen and Szkopiak ([60] and references within) reported that nitrogen was twice as effective as oxygen.

Oxygen contents as high as 0.41 wt.% increased the room temperature tensile strength of Nb from 276 MPa to 896 PMa and reduced the elongation from 30 to 10%. Contamination of Nb with oxygen increased its hardness [61] and caused embrittlement [62,63]. The latter has been attributed to screw dislocations moving through a repulsive field imposed by oxygen atoms, forming cross kinks and emitting excess vacancies in Nb which bind with oxygen and hinder dislocation motion [64].

Oxygen also affected the elevated temperature properties [62]. Tensile tests conducted on Nb with varying oxygen concentrations (10, 200 and 4300 wppm) revealed brittle failures below 400 °C for oxygen concentration of 0.43% [65]. At higher temperatures ductile failures were produced. The amount of ductility exhibited by Nb−O alloys at elevated temperatures was sensitive to strain-rate. For example, Nb containing 0.15% oxygen exhibited a decrease in the reduction in area from 90% to 30% at 467 °C due to a change in strain rate of 5 × 10^−5^ to 2 × 10^−1^ s^−1^ [66].

The DBTT of Nb depends on solute additions and increases with oxygen concentration as does the yield strength [67]. Interstitial elements have a significant effect on the DBTT, in that it can be raised as these impurities are increased. This trend in the data indicates that the interstitials progressively cause embrittlement and that the relative order of embrittlement is hydrogen (which is most potent), followed by oxygen and carbon (which is least potent). The effect of nitrogen is difficult to separate primarily because of uncertainties as to whether the solubility limit has been exceeded; however, based on very limited data, it appears to be more embrittling than either carbon or oxygen [68].

The sensitivity of the group V bcc metals to contamination with oxygen is greater compared with the group VI bcc metals Mo, W [69]. Contamination in air-reacted niobium, was similar to that in oxygen-reacted niobium, suggesting that oxygen is the primary diffusing contaminant [70]. Alloying Nb with Mo reduced the oxygen solubility, whereas alloying with Ti or Zr, respectively, increased and decreased it [71]. Contamination with hydrogen affected the shear moduli C’ = (C_11_ – C_12_)/2 and C_44_ and the bulk (K) and Young’s (E) moduli of V, Nb and Ta (group V bcc metals), of which the C’ decreased, the C_44_ increased, the K remained nearly constant, whereas the E of polycrystalline V or Ta with random orientation decreased and that of Nb increased with increasing hydrogen contamination. The effect on the C’ of V was about four times the effect in Nb and Ta, whereas the change in C_44_ with hydrogen was greatest for Nb and weakest for Ta [72]. Contamination with oxygen resulted in a small increase in C_44_ and K for V, but in the case of Nb, the C’ did not change with O ≤ 0.6 at.%, compared with the significant change in the C_44_ with O ≤ 0.7 at.%, and both C’ and C_44_ increased, respectively, by 1% and 7% with O ≤10 at.%, which could be associated with precipitation of Nb oxide. Furthermore, the change in the C_44_ of Nb was similar to that caused by the hydrogen contamination [72]. Hydrogen contamination increased the Young’s modulus of all three group V bcc metals [73] and the increase in E_110_ was very significant for Nb [74]. Contamination of Nb with oxygen (about 0.35 at.%) did not cause noticeable changes in the C’ and C_44_ shear moduli [75].

Regarding solid solutions of Nb with other bcc metals, contamination with oxygen affects mechanical properties. For example, for Nb-V alloys the addition of 500-ppm nitrogen and 1500-ppm (by weight) oxygen to Nb−2V and Nb−4V (wt.%) alloys caused pronounced increases in the DBTT of (Nb,V,I)_ss_ where I = O or N. Nitrogen was found to be more potent than oxygen as a strengthener. The influence of both nitrogen and oxygen on the mechanical properties increased with increasing V content [76]. The affinity of Al, Cr, Hf, Ti and Zr for oxygen is high (for example Hf or Zr is used to scavenge oxygen in RM alloys [56]).

**Figure 10:**
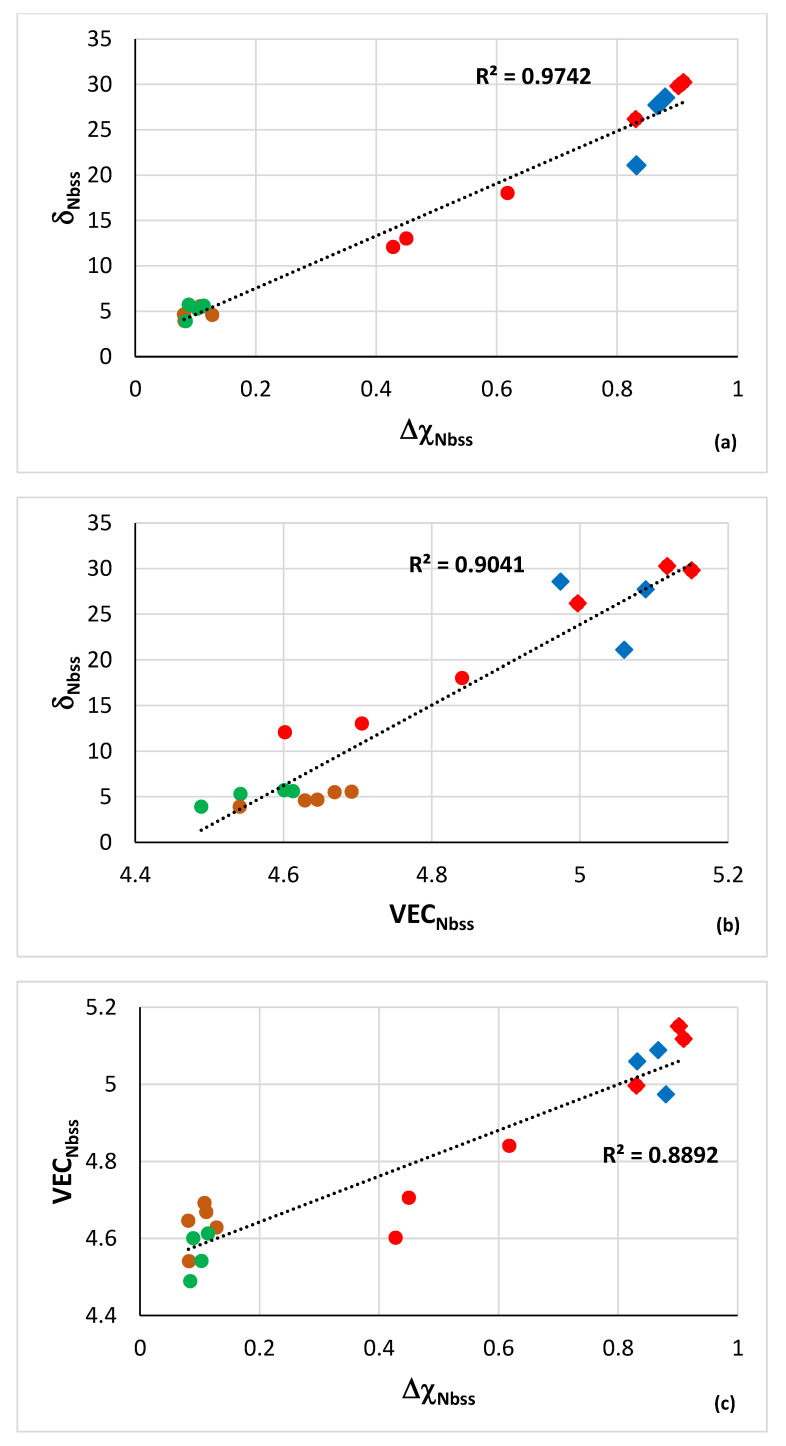

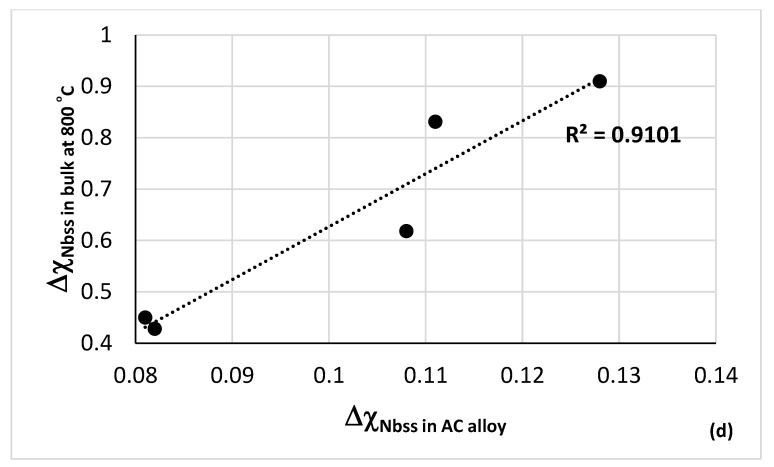
(**a**) δ_Nbss_ versus Δχ_Nbss_ (**b**) δ_Nbss_ versus VEC_Nbss_, (**c**) VEC_Nbss_ versus Δχ_Nbss_ and (**d**) Δχ of contaminated Nb_ss_ in the bulk of alloy after isothermal oxidation at 800 °C versus Δχ of “uncontaminated” Nb_ss_ in AC alloy. (**a**–**c**) colours: brown for Nb_ss_ in AC alloy, green for Ti rich Nb_ss_ in AC alloy, blue for Nb_ss_ in diffusion zone (DZ) formed at 800 °C, red for Nb_ss_ in bulk of alloy isothermally oxidised at 800 °C. Diamonds for CC/HE Nb_ss_. In each part the R^2^ value is for the linear fit of all the data. Data for the alloys NV1, NV2, NV5, ZX5 and ZX7. See Appendix A for nominal alloy compositions and references.

The bcc solid solution in RM(Nb)ICs, RM(Nb)ICs/RCCAs, RM(Nb)ICs/RHEAs, RHEAs and RCCAs is contaminated with oxygen, owing to the sensitivity of RMs on interstitial contamination and the presence of reactive elements in solution [3,10,13,16,17,18,19,42,77]. Contamination can be severe depending on alloying elements (e.g., see Figure 17 in [13]). There are limited data for contaminated Nb_ss_ and such data are available only for RM(Nb)ICs. These data show remarkable correlations between the parameters δ, Δχ and VEC. The data in Figure 10 are for “conventional” Nb_ss_ and Ti rich Nb_ss_ in AC alloys and for the diffusion zone (DZ) and bulk of alloys after isothermal oxidation at 800 °C for 100 h.

Figure 10 shows that δ_Nbss_ increases with increasing Δχ_Nbss_ or VEC_Nbss_ and that VEC_Nbss_ increases with increasing Δχ_Nbss_. The contaminated Nb_ss_ in the DZ is CC/HE Nb_ss_, whereas that in the bulk of oxidised alloys can be “conventional” Nb_ss_ or CC/HE Nb_ss_. The parameters of the solid solution in the AC alloys have the lowest values. There are also linear relationships between the parameters Δχ (Figure 10d), VEC and δ (figures not shown) of the contaminated Nb_ss_ in the bulk of alloy after isothermal oxidation at 800 °C versus the same parameter of “uncontaminated” Nb_ss_ in AC alloy that show the same trend as in Figure 10d, meaning the parameter of the former is higher the higher the parameter of the latter.

**Figure 11:**
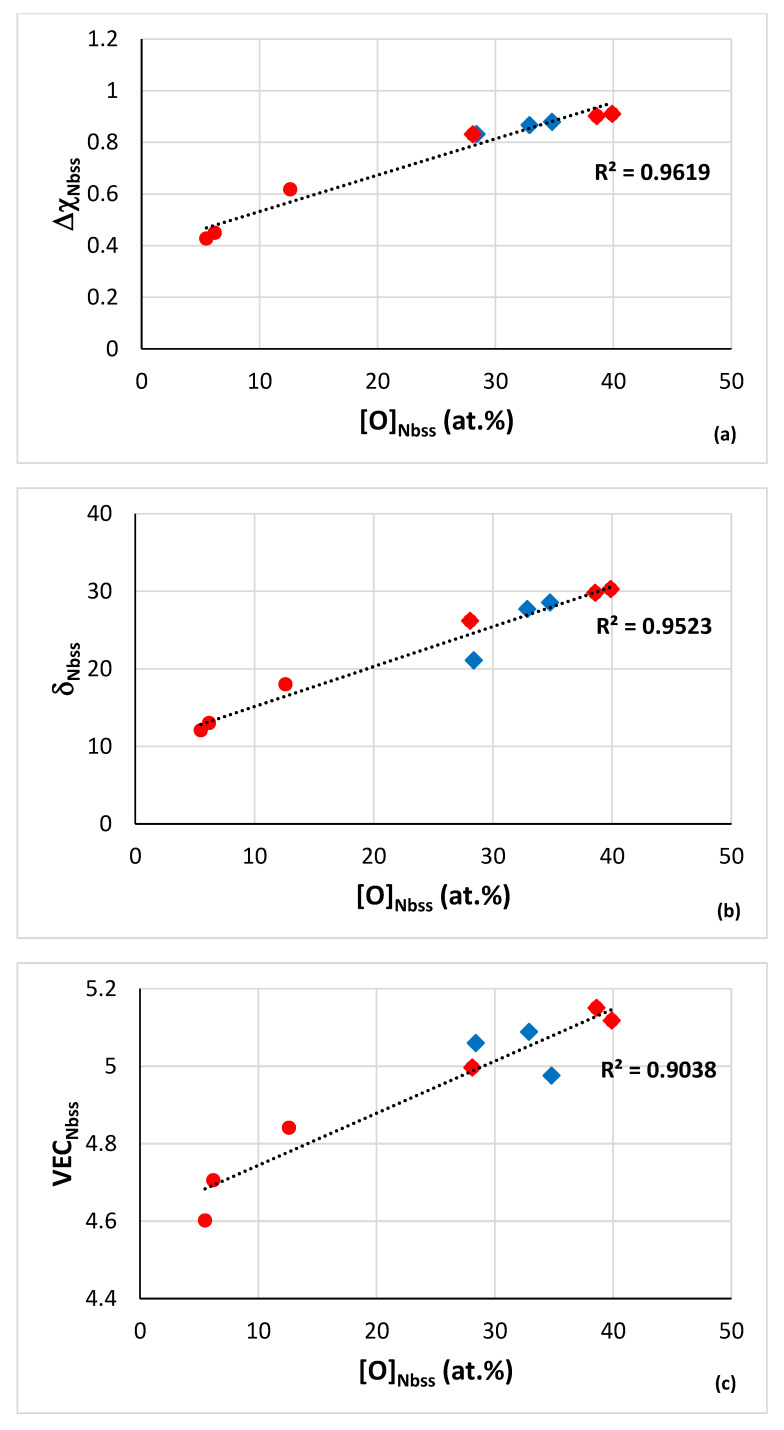
Relationships of the oxygen concentration in contaminated solid solutions in the diffusion zone and the bulk of alloys that were oxidised isothermally at 800 °C. Oxygen concentration (**a**) versus Δχ_Nbss_ (**b**) versus δ_Nbss_ and (**c**) versus VEC_Nbss_. Colours: blue for Nb_ss_ in diffusion zone (DZ) formed at 800 °C and red for Nb_ss_ in bulk of alloy isothermally oxidised at 800 °C. Diamonds for CC/HE Nb_ss_. In each part the R^2^ value is for the linear fit of all the data. Data for the alloys NV1, NV2, NV5, ZX5, ZX7. See Appendix A for nominal alloy compositions and references.

Remarkably, strong correlations also exist for the oxygen concentration in contaminated solid solutions in the diffusion zone and the bulk of isothermally oxidised alloys at 800 °C with the parameters Δχ_Nbss_ (Figure 11a), δ_Nbss_ (Figure 11b) and VEC_Nbss_ (Figure 11c), the values of which increase with increasing oxygen concentration in the solid solution. Note (i) that the chemical analysis data have been obtained using electron probe microanalysis [13,16,17,18] and (b) the strong correlation with the parameter Δχ_Nbss_. Moreover note that there are relationships between the concentrations of solutes in alloy and solid solution and the parameters Δχ_alloy_ and Δχ_ss_, respectively, which are key in alloy design using NICE [10].

The co-existence of CC/HE Nb_ss_ with “conventional” Nb_ss_ in most alloys [14] is further supported by the data in Figure 10, which also show that such relationships between the parameters δ, Δχ and VEC can be used in NICE to predict whether the microstructure of a designed alloys will consist of “conventional” Nb_ss_ and CC/HE Nb_ss_ and what the chemical compositions of such solid solutions would be. Furthermore, Figure 11 shows that contamination with oxygen affects all three parameters, which are related with atomic size, electronegativity and electron concertation in the valence band [10,28] and can account for changes in mechanical properties (creep, strength) and oxidation [9,10,14,17,18,32,33,34,42].

### 3.2. Effect of Contamination with Oxygen on Properties of the Solid Solution

#### 3.2.1. Hardness

Contamination of Nb with oxygen increases the Vickers hardness and yield strength of (Nb,O)_ss_ and also increases its DBTT (Section 3.1). Contamination of the Nb solid solution in RM(Nb)ICs, RM(Nb)ICs/RCCAs or RM(Nb)ICs/RHEAs would affect its mechanical properties, in particular its hardness/yield strength and Young’s modulus [3]. In each of these types of alloys and other RCCAs and RHEAs, for example those included in the review in [19], the contamination of the bcc solid solution will be different as it depends on the chemical composition of the solid solution, alloy condition (AC, HT) and environment of operation. For example, the contamination of the solid solution of the alloy NV1 was very sever, compared with other RM(Nb)ICs, see Figure 17 in [13]. I shall demonstrate the effects of contamination of bcc solid solution with oxygen on properties using data for the Nb_ss_ in the RM(Nb)IC alloy NV1.

Why the alloy NV1? The high vol.% Nb_ss_ (about 81%) in this alloy made feasible the measurement of the nanohardness of the Nb_ss_ using nanoindentation, as discussed in [16]. Furthermore, the solute additions included key solute elements in metallic UHTMs, namely Al, Cr, Hf, Nb, Ti and V as well as Si and Sn.

The alloy NV1 was heat treated at 1500 °C for 100 h in a Ti-gettered argon atmosphere [16]. Contamination of the alloy could not be avoided even under these HT conditions. The nanohardness (nanoH, GPa) and the reduced elastic modulus E_r_ (GPa) of the Nb_ss_ of NV1-HT was measured from the surface of the heat-treated specimen to 2000 μm below the surface. A Hysitron TriboScope nano-mechanical testing system was used [16], with 8000 μN indenter load. A 4 × 4 testing array was created over a 50 μm × 50 μm area, 16 indents per area with 10 μm spacing. Data were collected from the surface and areas below it every 40 μm to a depth of 400 μm, then at 470 μm, then every 100 μm to 770 μm depth, and then at 940, 1070, 1220, 1390, 1590 and 2000 μm [78]. The microstructure of NV1-HT was shown in Figure 2 in [16].

**Figure 12:**
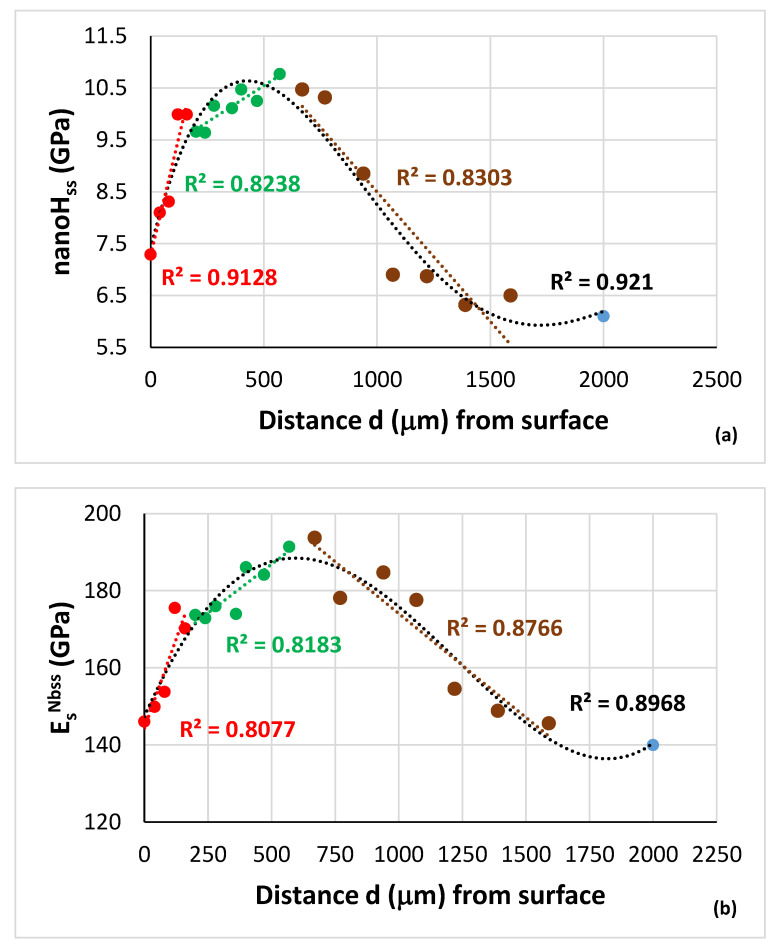

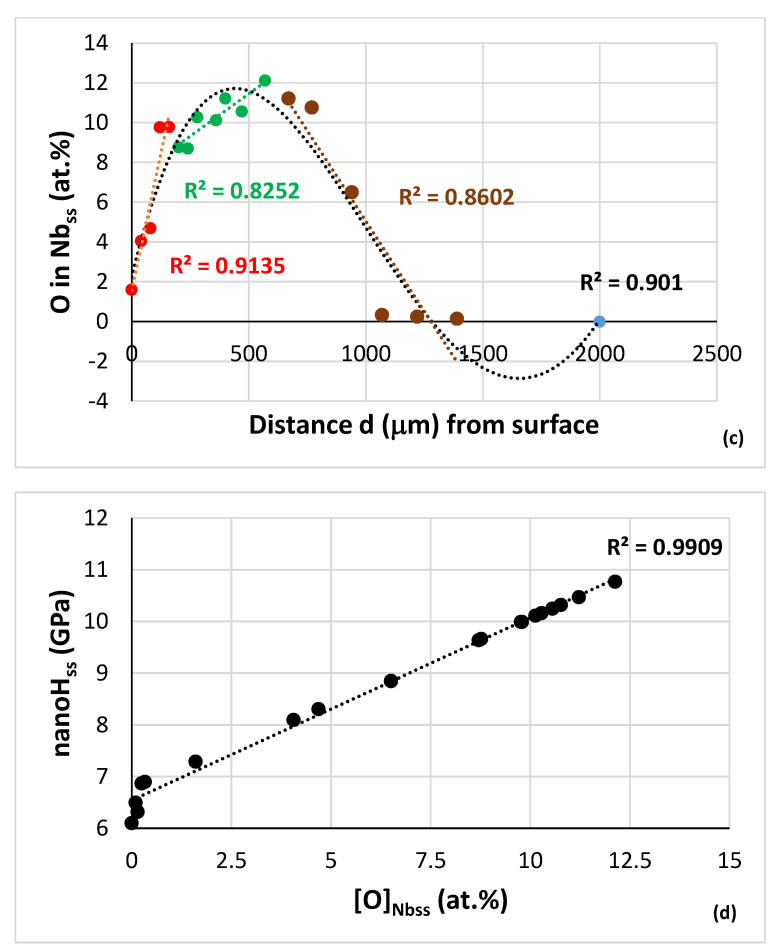
(**a**) Average nanohardness, (**b**) average Young’s modulus and (**c**) average oxygen concentration of the Nb_ss_ in NV1-HT (1500 °C/100 h) as a function of distance from the surface of heat treated specimen. In each part, all the data fit to a 4th order polynomial (see text) with R^2^ values 0.921, 0.8968 and 0.9016, respectively, for (**a**–**c**). (**d**) Nanohardness of Nb_ss_ versus oxygen content.

The data in Figure 12a show that the nanoH_ss_ increased to a maximum value in the area that was 570 μm below the surface, and then decreased to the “bulk” value of the HT specimen (blue data point). In Figure 12a, all the data fit to the 4th order polynomial nanoH_ss_ = −3 × 10^−12^d^4^ + 2 × 10^−08^d^3^ − 3 × 10^−05^d^2^ + 0.0175d + 7.4127 with R^2^ = 0.921. First, there was a rapid increase in nanohardness (red data points, R^2^ for linear fit of data) to about 120 μm, then the change in nanohardness with distance decreased (green data, R^2^ for linear fit of data) and the nanohardness reached its maximum value in the area 570 μm below the surface, then the nanohardness decreased with distance from 570 μm to about 1220 μm (brown data points, R^2^ for linear fit of data) followed with minor changes for distances greater than 1590 μm below the surface. A similar hardness profile to that shown in Figure 12a was reported in [70] for contamination of Nb with oxygen (i.e., for (Nb,O)_ss_) after 1.62 h at 1000 °C, where the depth of contamination was at least 760 μm.

In Figure 12a the surface nanohardness is 7.29 GPa or 743.3 HV and corresponds to microhardness (microH) 548.2 HV based on the relationship microH_Nbss_ = 0.7357 × nanoH_Nbss_ (see [16]), whereas the maximum nanohardness of 10.77 GPa or 1098 HV at 570 μm below the surface corresponds to microhardness 807.9 HV. In the area 2000 μm below the surface the nanohardness was 6.1 GPa or 622 HV and corresponds to microH_Nbss_ = 457.6 HV. This is lower than the hardness of the solid solution (523 HV) reported in [16], where the area of hardness measurement below the surface was not recorded. The surface hardness and the maximum hardness of the alloyed and contaminated with oxygen Nb_ss_ in NV1-HT below the surface, respectively, was more than 7 and 10 times that of “uncontaminated” Nb. Up to 15 times increase in hardness has been reported for Nb contaminated with 16 at.% C, i.e., for (Nb,C)_ss_ [79].

The hardness of the contaminated with oxygen Nb_ss_ in NV1-HT at the surface (548 HV), and 570 μm below the surface (808 HV) was higher than the hardness of the (uncontaminated?) single bcc solid solution phase RHEAs HfMoTaTiZr (542 HV), MoNbTaVW (535 HV), HfMoNbTaTiZr (505 HV), MoNbTaV (504 HV), NbTaVW (493 HV), MoNbTaW (454 HV), NbTaTiVW (447 HV), MoNbTaTiV (443 HV), TaNbHfZrTi (409 HV), HfNbTaTiZr (390 HV), NbTiVZr (335), NbTaTiV (298 HV) [80].

The EPMA analyses of Nb_ss_ grains in areas about 600 μm below the surface gave the average composition of the contaminated Nb_ss_ as 54.1(±4, 49.9–58.7)Nb–17.6(±3.2, 13.3–21.8)Ti–0.6(±0.3, 0–0.9)Si–5(±0.2, 4.8–5.5)Al–2.7(±0.5, 2.1–3.5)Cr–5.6(±0.7, 4.9–6.6)V–2.2(±0.5, 1.2–2.7)Sn–0.2(±0.1, 0–0.4)Hf–12.1(±2, 8.8–15.2)O, where in parenthesis is given the standard deviation and the minimum and maxim analysis value. There was no second phase precipitation in the solid solution. The oxygen concentration of 12.1 at.% and Figure 11b give δ_ss_ = 16.24. The ascending part of the HV_ss_ versus δ_ss_ data (brown data) in Figure 7 in [3] gives microH_ss_^600 μm^ = 797 HV that corresponds to nanoH_ss_^600 μm^ = 1083 HV or nanoH_ss_^600 μm^ = 10.62 GPa. The highest measured nanohardness of the Nb_ss_, which was for the area 570 μm below the surface (see above), and Figure 7 in [3] give δ_ss_ = 16.66 and from Figure 11b we obtain the oxygen content of 12.96 at.%. Both oxygen concentrations are higher than the maximum solubility of oxygen in Nb (9 at.% at 1915 °C) according to the Nb-O binary phase diagram [69] and would suggest that the hardness increased with distance below the surface to the area where the Nb_ss_ most likely became saturated with oxygen. Note (i) that the 9 at.% O solubility is for the (Nb,O)_ss_, (ii) that the maximum solubility of oxygen in the Nb_ss_ of NV1 is not known, (iii) that the Nb_ss_ in NV1 was heavily alloyed and its contamination was more severe compared with the Nb_ss_ in other RM(Nb)ICs (see Figure 17 in [13]) and (iv) that no precipitation of a second phase in the Nb_ss_ was observed in the areas below the surface where nanoindentation was performed.

NICE has demonstrated how the relationships between parameters of alloys and their phases and between parameters and properties of alloys and their phases can assist the alloy designer to design/select new alloys worthy of R&D work [9,10,14]. Below it will be shown that it is possible to use such relationships to understand/predict how contamination with oxygen or alloying with boron affect properties of the solid solution.

**Figure 13:**
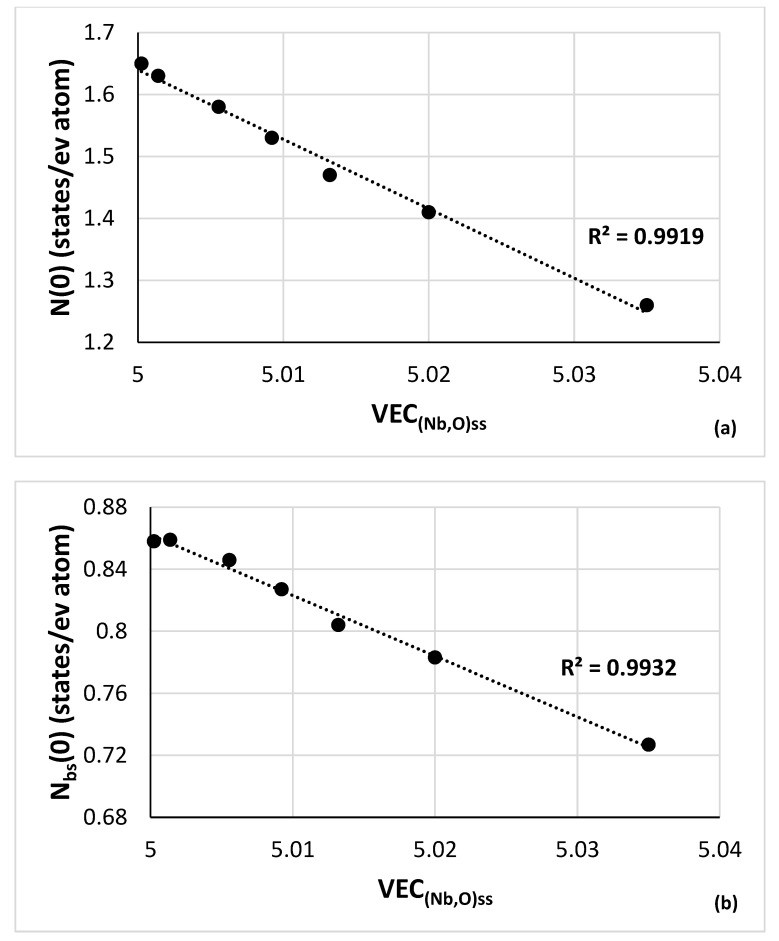
Data for Nb contaminated with oxygen. (**a**) density of electronic states at the Fermi level N(0) and (**b**) “band structure” density of states N_bs_(0) versus the parameter VEC of the (Nb,O)_ss_.

Owing to contamination of Nb with oxygen the hardness and the lattice parameter of the (Nb,O)_ss_ increase. This is well documented in the literature, for example see [60] and references within, and [61]. The effect of oxygen contamination on the hardness of Nb is given with linear relationships of the form HV_(Nb,O)ss_ = A[O] + HV°_Nb_ where [O] is concentration of oxygen and HV°_Nb_ is the hardness of “uncontaminated” “pure” Nb. In the literature the values of the constant A and HV°_Nb_ differ because they depend on the purity of the starting “uncontaminated” Nb, the method of preparation of the (Nb,O)_ss_ and the analysis method used. For example, when the main impurities of the Nb were Ta (860 ppm) and W (460 ppm) Kotch et. al. gave HV_(Nb,O)ss_ = 90.903[O] + 77.566 with R^2^ = 0.9762. The lattice parameter of the contaminated Nb was given by the same researchers as α_o_^(Nb,O)ss^ (Å) = 0.0039[O] + 3.3 with R^2^ = 0.9622 [81]. Furthermore, they reported that the contamination of Nb with oxygen decreased the density of electronic states at the Fermi level N(0) and the “band structure” density of states N_bs_(0) [81], both of which correlate well with the parameter VEC of the (Nb,O)_ss_ (Figure 13a,b). The importance of the parameter VEC for the properties (oxidation, creep) of RM(Nb)ICs was discussed in [10].

##### Boron Free RM(Nb)ICs

For boron free KZ series alloys (KZ series alloys are RM(Nb)ICs based on Nb-24Ti-18Si (at.%, nominal) with addition of Al, Cr individually or simultaneously, for example the alloys KZ4, KZ5 and KZ7, or with simultaneous addition of Al, Cr and Ta, for example the alloy KZ6, see Appendix A for nominal compositions) the hardness of the Nb_ss_ depends on δ with a linear relationship of the form HV_ss_ = aδ + b of which the constants a and b are both positive (for example, see the ascending data (brown data points) in Figure 7 in [3]). The values of these constants change when Sn or Ge is present in the alloy with/without Hf but they are still positive. The constant b is the hardness of Nb_ss_ for which the type of solute elements and their concentrations give δ = 0 (solute additions and contamination with oxygen will change the lattice parameter). The parameter δ of the Nb_ss_ depends on oxygen concentration with a linear relationship of the form δ = c[O] + d (Figure 11b) where both the constants c and d are positive and [O] is the concentration of oxygen in the Nb_ss_. The constant d is the value of the parameter δ of the “uncontaminated” Nb_ss_.

For a specific alloy 1, HV_ss1_ = a_1_δ_1_ + b_1_ and δ_1_ = c_1_[O] + d_1_. Thus HV_ss1_ = a_1_(c_1_[O] + d_1_) + b_1_ = a_1_c_1_[O] + a_1_d_1_ + b_1_ or HV_ss1_ = A_1_[O] + B_1_, where A_1_ = a_1_c_1_ and B_1_ = a_1_d_1_ + b_1_. Both A_1_ and B_1_ are positive. The value of A_1_ will be deferent from the value of A for (Nb,O)_ss_ (see previous section), and will depend on the solute elements in Nb_ss_, which sequentially affect the severity of contamination of the solid solution (see Figure 17 in [13]). In other words, the value of A_1_ will depend on the specific RM(Nb)IC, RM(Nb)IC/RCCA or RM(Nb)IC/RHEA being considered. For the specific alloy 1 the hardness of its Nb_ss_ for zero [O], i.e., the value of B_1_, is made of two parts, one (the constant b_1_) is the hardness of a Nb_ss_ with the same solute elements and concentrations that give δ = 0 and the other part depends (i) on how changes in atomic size, owing to alloying additions and their concentrations (excluding oxygen contamination) affect hardness (the constant a_1_) and (ii) on how oxygen contamination affects atomic size (the constant d_1_).

For the Nb_ss_ of the alloy NV1-HT, the hardness is HV_ss NV1-HT_ = A_ss NV1-HT_[O] + B_ss NV1-HT_. If we were to assume that B_ss NV1-HT_ is the average of the measured microhardness values of the Nb_ss_ in the bulk of NV1-HT given in [16] and in [82] and [78] (i.e., B_ss NV1-HT_ = 507 HV), and take into account the measured oxygen content of Nb_ss_ in NV1-HT below the surface (see previous section) and the nanohardness data for different areas below the surface (Figure 12a), we can calculate the oxygen concentration of the Nb_ss_ with distance below the surface of NV1-HT. This is shown in Figure 12c. Similarly with the other two parts of Figure 12, all the data for oxygen concentration as a function of distance below the surface fit to the 4th order polynomial [O]_Nbss_ (at.%) = −4 × 10^−12^d^4^ + 3 × 10^−08^d^3^ − 8 × 10 ^−05^d^2^ + 0.049d + 2.2136 with R^2^ = 0.901. In Figure 12c, the blue data point corresponds to Nb_ss_ in the bulk. The hardness of the solid solution increased with oxygen contamination. The R^2^ values were 0.9909, 0.9907 and 0.9792, respectively, for the linear fit of the nanoH_ss_ data versus [O], [O]^2/3^ and [O]^0.5^ (at.%). The best fit of the data is shown in Figure 12d.

**Figure 14:**
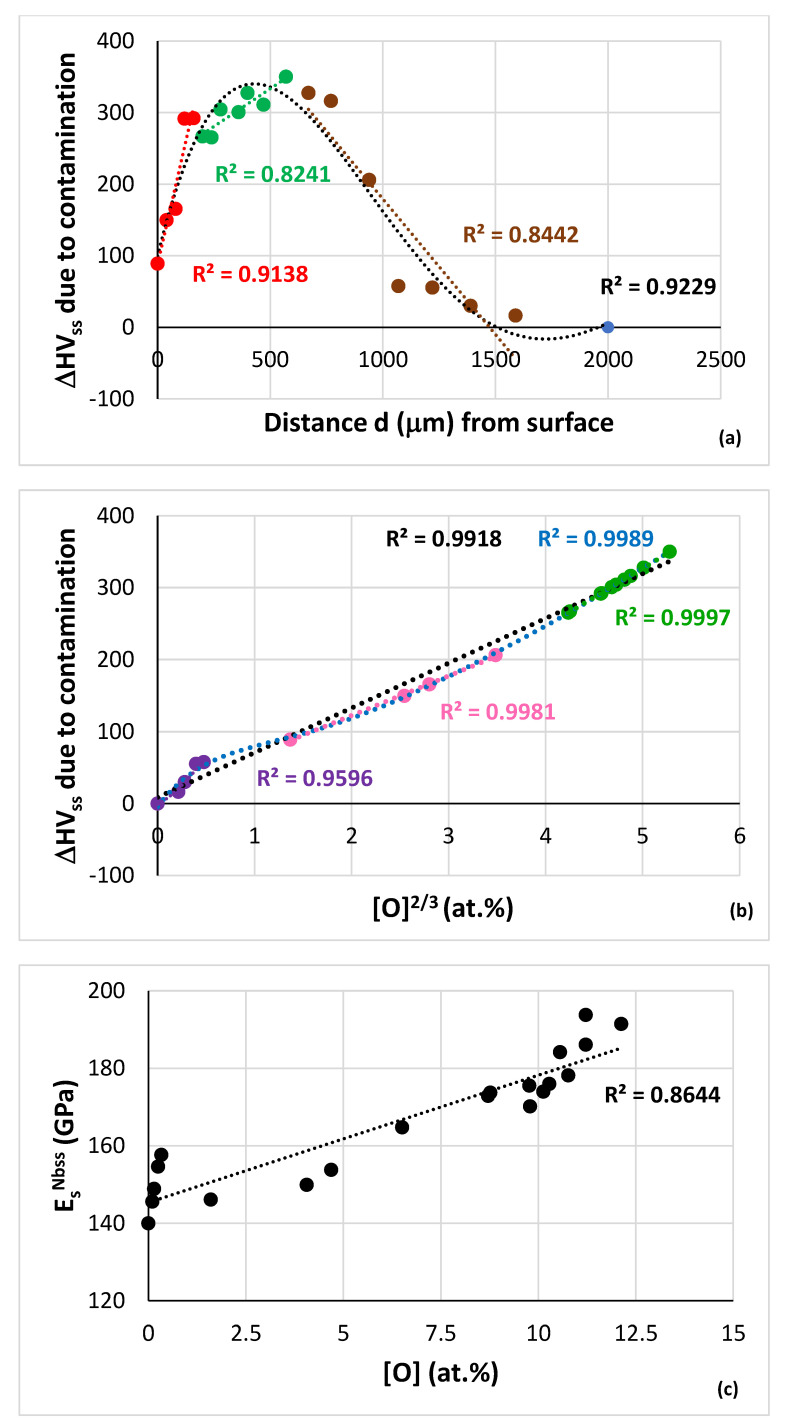

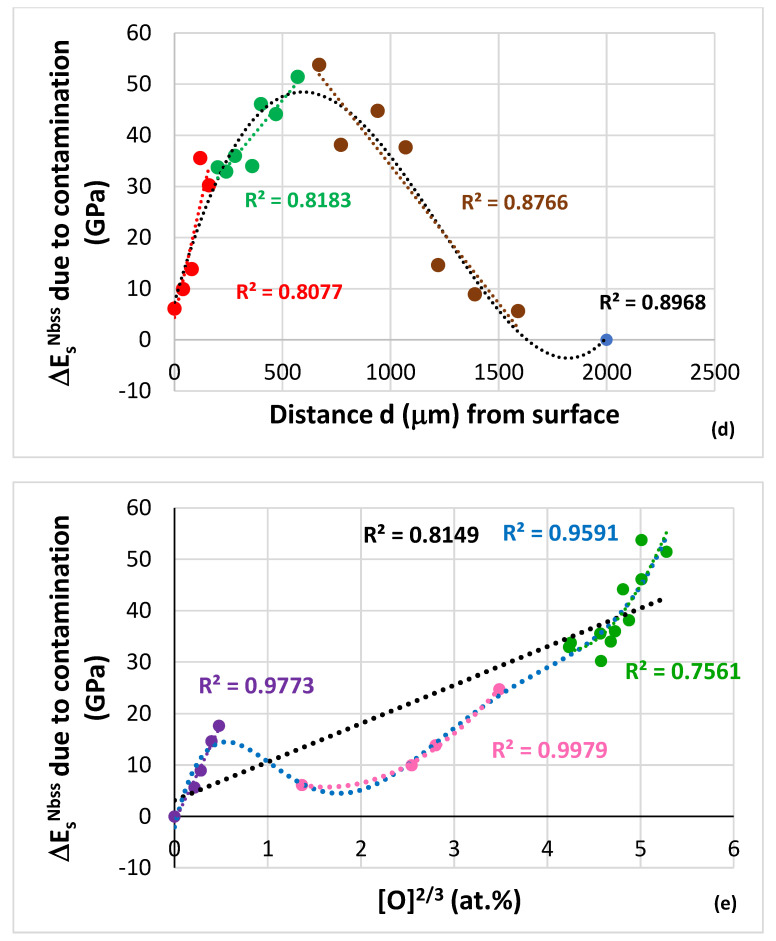
Data for the bcc solid solution Nb_ss_ in the alloy NV1-HT. (**a**,**b**) change in solid solution hardness, respectively, with distance below the surface and with oxygen contamination. (**c**,**e**) dependence on oxygen contamination, respectively, of the nanoindentation Young’s modulus and its change with oxygen concentration. (**d**) Change in nanoindentation Young’s modulus because of contamination with oxygen with distance below the surface. In (**b**,**e**) the black and blue dashed lines, respectively, are for linear and polynomial fit of all data.

For the alloy NV1, if we assume that the solid solution at 2000 μm below the surface was uncontaminated we can obtain the contribution to solid solution hardening from the alloying additions as HV_Nbss_^2000 μm^ − HV°_Nb_. The contribution of solute additions to hardening depends on the value of HV°_Nb_, it would be about 437.5 HV if we take HV°_Nb_ as the average of the values in the Table 1 in [60] or about 430 HV if we take HV°_Nb_ = 77.6 from [81]. We can also calculate the change in hardness of the Nb_ss_ (ΔHV) due to contamination with oxygen (ΔHV = HV_Nbss contaminated_ − HV_Nbss_^bulk^) with distance d below the surface. This is shown in Figure 14a, where the data fit to a 4th order polynomial ΔHV = −2 × 10^−10^d^4^ + 1 × 10^−06^d^3^ − 0.0022d^2^ + 1.2977d + 99.418 with R^2^ = 0.9229. The contribution to hardening of the solid solution due to contamination with oxygen increased with the concentration of the latter in the Nb_ss_. The R^2^ values were 0.9916, 0.9918 and 0.9805, respectively, for the linear fit of ΔHV_ss_ data versus [O], [O]^2/3^ and [O]^0.5^ (at.%). The best linear fit of the data is shown in Figure 14b. Note that all the data in Figure 14b fit to a 6th order polynomial ΔHV_ss_ = −0.1603x^6^ + 3.0517x^5^ − 23.073x^4^ + 87.252x^3^ − 163.54x^2^ + 180.57x − 4.4811 with R^2^ = 0.9989, shown with blue dashed line, and that at low oxygen contents the increase in ΔHV_ss_ is parabolic (R^2^ = 0.9596), followed by linear increases (R^2^ = 0.9981 and R^2^ = 0.9997) with increasing ΔHV_ss_/[O]^2/3^ as the severity of contamination with oxygen increased, in agreement with [60].

##### Boron Containing RM(Nb)ICs

For boron containing KZ series alloys (for “definition” of these alloys see the previous section) the hardness of the solid solution also is given by a liner relationship of the form HV_ss_ = aδ + b, where a < 0 and b > 0 (see the descending part of the data (green data points) in Figure 7 in [3]). Currently, there are no data for the change in the parameter δ with oxygen concentration in the Nb_ss_. Let us assume (i) that a linear relationship of the form δ = c[O] + d applies for the dependence of δ on contamination and (ii) that for an alloy 1 the hardness of its Nb_ss_ increases with oxygen contamination, i.e., the equation HV_1_ = A_1_[O] + B_1_ applies, or HV_ss1_ = a_1_c_1_[O] + a_1_d_1_ + b_1_. Given that a_1_ < 0, the first term would be negative if c_1_ were to be positive. Thus, based on the aforementioned assumptions, we conclude that c_1_ must be negative, in other words the parameter δ of the solid solution would decrease as the [O] concentration increases. This must be tested experimentally. The last two terms (i.e., B_1_ = a_1_d_1_ + b_1_) give the hardness of the “uncontaminated” solid solution, i.e., Nb_ss_ with [O] = 0 at.%. Similarly with the boron free alloys, the value of B_1_ is made of two parts, one (the constant b_1_) is the hardness of a Nb_ss_ with the same solute elements and concentrations that give δ = 0 and this part is positive, and the other part, which in this case is negative, depends (i) on how changes in atomic size affect hardness (the constant a_1_ < 0) owing to the alloying additions and their concentrations (excluding oxygen contamination) and (ii) on how oxygen contamination affects atomic size (the constant d_1_ > 0).

**Figure 15:**
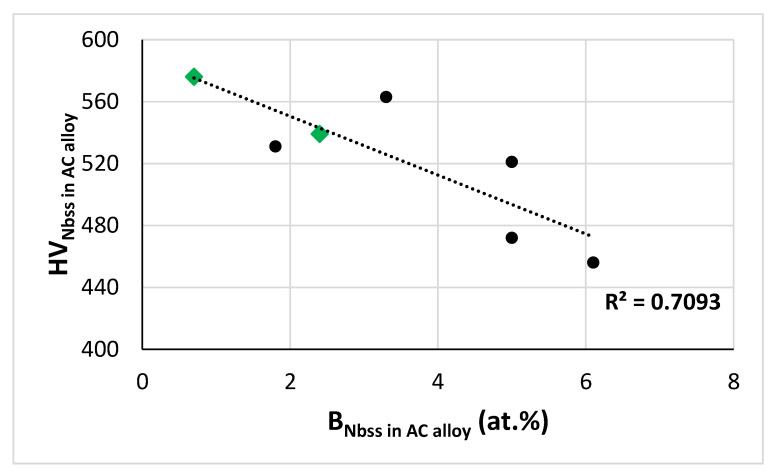
Hardness of the Nb_ss_ in AC boron containing KZ series alloys versus the B concentration of the solid solution. Data for the alloys TT1, TT2, TT3, TT4, TT5, TT7 and TT8. Green diamonds for Nb_ss_ in RM(Nb)ICs/RCCAs. All data R^2^ = 0.7093.

However, in the case of boron containing KZ series alloys, the parameter δ of the Nb_ss_ depends on its boron concentration, as shown in Figure 7a, and the hardness of the solid solution decreased with increasing B concentration (Figure 15). In other words, for these alloys the experimental evidence gives HV = C[B] + D, where C < 0, and D > 0 (Figure 15) and δ = e[B] + f, where e > 0 and f > 0 (Figure 7a). Thus, for a boron containing alloy 1, HV_ss1_ = C_ss1_[B] + D_ss1_, HV_ss1_ = a_1_δ + b_1_ (where a_1_ < 0, see previous paragraph) or HV_ss1_ = a_1_(e_1_[B] + f_1_) + b_1_ or HV_ss1_ = a_1_e_1_[B] + a_1_f_1_ + b_1_. Therefore, C_ss1_ = a_1_e_1_, i.e., the constant C_ss1_ is negative, in agreement with the experimental data (Figure 15). The value of D_ss1_ = a_1_f_1_ + b_1_ is made of two parts, one (the constant b_1_) is the hardness of a Nb_ss_ with the same solute elements and concentrations that give δ = 0 and this part is positive, as was the case for B_1_ (see above), and the other part, which in this case is negative, depends (i) on how changes in atomic size affect hardness (the constant a_1_ < 0) owing to the alloying additions and their concentrations (excluding oxygen contamination), as was the case for B_1_, and (ii) on how alloying with boron affects atomic size (the constant f_1_ > 0), differently with B_1_.

#### 3.2.2. Young’s Modulus

The Young’s modulus E_s_ (GPa) of the Nb_ss_ was calculated using the data from the nano-indentation experiments (see Section 3.2.1) and Equation (1)

(1) $$E_{s}=\frac{E_{r}E_{i}(1-\nu_{s}{}^{2})}{E_{i}-E_{r}(1-\nu_{i}{}^{2})}$$

where *E_s_* and *ν_s_* are the Young’s modulus and Poisson’s ratio of the phase, and *E_i_*, *ν_i_* are the parameters for the Berkovich indenter [83]. For the calculation of *E_s_* the values of *E_i_* and *ν_i_* that were given in the TriboScope manual [84] as 1140 GPa and 0.07, respectively, were used and the *ν_s_* was 0.38 [46,85]. Data for *E_s_* are shown in Figure 12b.

The data showed that E_r_^Nbss^ and E_s_^Nbss^ increased to maximum values in the area 570 μm below the surface, and then decreased to the “bulk” of the HT specimen (blue data point). All the data fit to 4^th^ order polynomials, as follows: E_r_^Nbss^ = 6 × 10^−12^d^4^ + 2 × 10^−08^d^3^ – 0.0001d^2^ + 0.1284d + 149.81 with R^2^ = 0.8972 (figure not shown) and E_s_^Nbss^ = 6 × 10^−12^d^4^ + 3 × 10^−08^d^3^ − 0.0002d^2^ + 0.1501d + 147.33 with R^2^ = 0.8968 (Figure 12b). First, there is a rapid increase in E_s_ from 146 GPa at the surface to 175 GPa about 120 μm below the surface (red data points, the R^2^ value is for linear fit of data), then the change in E_s_ with distance decreases (green data, the R^2^ value is for linear fit of data) and reaches its maximum value of 194 GPa in the area 670 μm below the surface, then the E_s_ decreases with distance to about 155 GPa at 1220 μm (brown data points, the R^2^ value is for linear fit of data) followed with minor changes for distances greater than 1590 μm below the surface to about 140 GPa in the bulk.

The values of the Young’s modulus of the alloyed and contaminated with oxygen Nb_ss_ in NV1-HT at 400 to 570 μm below the surface approached that of unalloyed γNb_5_Si_3_ [46]. Significant increase in the Young’s modulus of Nb owing to interstitial contamination has been reported for Nb contaminated with 16 at.% C (i.e., for (Nb,C)_ss_), where the increase was up to three times the Young’s modulus of “uncontaminated” Nb [79].

The Young’s moduli of the Nb_ss_ at the surface and the bulk of NV1-HT were higher than those of the (uncontaminated?) single phase solid solution RHEAs TiZrNbMo (142 GPa), TiZrNbMoV (141 GPa), MoNbTaTiV (139 GPa), TiZrVNb (121 GPa), NbTaTiV (117 GPa), TaNbHfZrTi (104 GPa), TiZrHfNbCr (104 GPa), TiZrHfNbV (95 GPa) anc TiZrHfNb (89 GPa), and the maximum Young’s modulus (194 GPa) was lower than that of the (uncontaminated?) single phase solid solution RHEAs NbMoTaW (229 GPa), VnbMoTaW (205 GPa) and AlMoNbV (197 GPa) [86].

**Figure 16:**
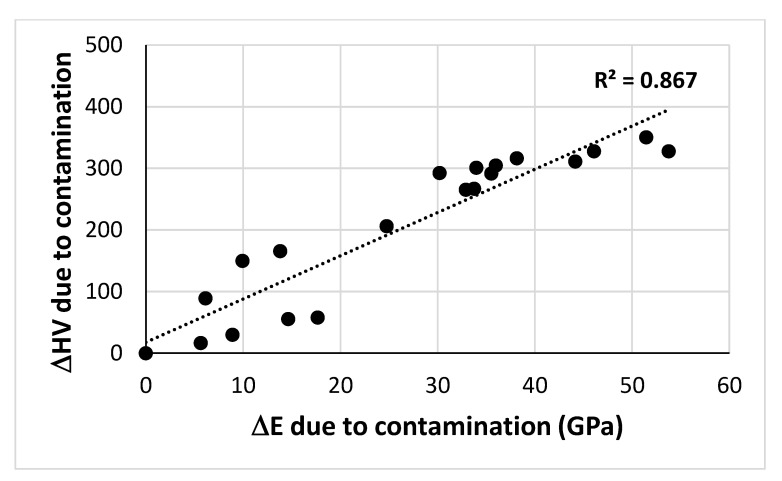
Change in Vickers hardness versus the change in Young’s modulus of the solid solution due to contamination with oxygen. The R^2^ value is for the linear fit of all data.

The Young’s modulus of the Nb_ss_ in NV1-HT increased with oxygen contamination. The R^2^ values were 0.8644, 0.8149 and 0.7815, respectively, for the linear fit of E_s_ data versus [O], [O]^2/3^ and [O]^0.5^ (at.%). The best linear fit of the data is shown in Figure 14c. If we take the Young’s modulus of uncontaminated and pure Nb as E°_Nb_ = 101.9 GPa [46] and assume that the solid solution at 2000 μm below the surface was uncontaminated, we can obtain the contribution to the Young’s modulus from the alloying additions (about 38.1 GPa) and then we can calculate the change in the Young’s modulus of the Nb_ss_ (ΔE_s_) due to contamination with oxygen with distance below the surface, as shown in Figure 14d, where ΔE_s_ = 6 × 10^−12^d^4^ + 3 × 10^−08^d^3^ − 0.0002d^2^ + 0.1501d + 7.3296, with R^2^ = 0.8968. The R^2^ values were 0.8644, 0.8149 and 0.7815, respectively, for the linear fit of ΔE_s_ data versus [O], [O]^2/3^ and [O]^0.5^ (at.%), but the best fit to 6th order polynomial was for ΔE_s_ versus [O]^2/3^, which is shown with the dashed blue line in Figure 14e for which ΔE_s_ = −0.0951x^6^ + 2.0575x^5^ − 16.628x^4^ + 62.854x^3^ − 109.2x^2^ + 73.741x − 2.07 with R^2^ = 0.9591. Note that at low contamination level the increase in ΔE_s_ was linear (R^2^ = 0.9773) and was followed with parabolic increases (R^2^ = 0.9979 and R^2^ = 0.7561) as the severity of contamination increased. As shown in the Figure 16 there is a linear relationship between the change in Vickers hardness (ΔHV) and the change in Young’s modulus (ΔE) of the solid solution due to contamination with oxygen.

## 4. Summary

In this paper the stability of CC/HE solid solutions and the contamination with oxygen of solid solutions in (RM(INb)ICs), RM(Nb)ICs/RCCAs and RM(Nb)ICs/RHEAs was studied. “Conventional” solid solutions were compared with CC/HE ones. “Conventional” Nb_ss_ can be Ti rich only in AC alloys, and Ti rich Nb_ss_ is not observed in HT alloys. In B containing alloys the Ti rich solid solution is usually CC/HE Nb_ss_. The CC/HE Nb_ss_ is stable after heat treatment in alloys with simultaneous addition of Mo, W with Hf, Ge and Sn. There is a strong correlation between the δ parameters of “conventional” and CC/HE Nb_ss_ with the B or the Ge+Sn concentration in the solid solution. Similarities and differences between relationships of other solutes in alloys with B or Ge+Sn addition were noted and their implications for alloy design were discussed.

The CC/HE Nb_ss_ has low Δχ, VEC and Ω and high ΔS_mix_, |ΔH_mix_| and δ parameters and is formed in alloys that also have high entropy of mixing. These parameters were compared with those of single-phase solid solution HEAs and differences in the values of ΔH_mix_, δ, Δχ and Ω, and similarities in the values of ΔS_mix_ and VEC were discussed. Relationships between the alloy and “conventional” solid solution parameters in NICE also apply for CC/HE Nb_ss_. The parameters δ_ss_ and Ω_ss_, and VEC_ss_ and VEC_alloy_ can differentiate between types of alloying additions and their concentrations and are key regarding the formation or not of CC/HE Nb_ss_.

After isothermal oxidation at a pest temperature (800 °C/100 h) the contaminated with oxygen Nb_ss_ in the diffusion zone is CC/HE Nb_ss_, whereas the solid solution in the bulk of the oxidised alloys can be “conventional” Nb_ss_ or CC/HE Nb_ss_. The parameters of “uncontaminated” and contaminated with oxygen solid solutions are linked with linear relationships. There are strong correlations between the oxygen concentration in contaminated solid solutions in the diffusion zone and the bulk of isothermally oxidised alloys at 800 °C with the parameters Δχ_Nbss_, δ_Nbss_ and VEC_Nbss_, the values of which increase with increasing oxygen concentration in the solid solution.

Correlations between oxygen content and the parameters δ, Δχ and VEC showed that the effects of interstitial contamination on properties can be understood and/or described with all three parameters. The boron content on the other hand correlates only with δ.

The effects of contamination with oxygen of the near surface areas of a heat-treated RM(Nb)IC with high vol.% Nb_ss_ on the hardness and Young’s modulus of the solid solution, and contributions to the hardness of the Nb_ss_ in B free or B containing KZ series alloys were discussed. The hardness and Young’s modulus of the bcc solid solution increased linearly with its oxygen concentration and the change in hardness and Young’s modulus due to contamination increased linearly with [O]^2/3^.

## 5. Suggestions for Future Research

In RM(Nb)ICs/RCCAs, the CC bcc solid solutions with Ge+Sn and Al, Cr, Hf, Mo, Ti and W additions are (i) stable (Figure 1) and (ii) Si free [33,34]. It is suggested (a) that single phase bcc solid solution RCCAs or RHEAs with above elements would be stable at high temperatures. Furthermore, given that Ge+Sn with Al, Cr, Hf, Mo and Ti improve oxidation at pest and high temperatures [32,33,34,87] it is suggested (b) that these RCCAs or RHEAs would also be oxidation resistant. For B containing RM(Nb)ICs, RM(Nb)ICs/RCCAs, RM(NB)ICs/RHEAs, RCCAs and RHEAs it is suggested to study the contamination with oxygen of their solid solutions and to find out if there is a relationship between the parameter δ and the oxygen concentration, see Boron Containing RM(Nb)ICs in Section 3.2.1.

## Data Availability

All the data for this work is given in the paper, other data cannot be made available to the public.

## Appendix A

**Table A1:** Nominal compositions (at.%) of reference alloys used in this work.

Alloy	Nb	Ti	Si	Al	B	Hf	Cr	Mo	Ta	V	W	Fe	Ge	Sn	Ref.
EZ5	43	24	18	5	-	5	-	-	-	-	-	-	-	5	[15]
EZ6	43	24	18	-	-	5	5	-	-	-	-	-	-	5	[15]
EZ8	38	24	18	5	-	5	5	-	-	-	-	-	-	5	[15]
JG3	46	24	18	5	-	-	5	2	-	-	-	-	-	-	[87]
JG6	36	24	18	5	-	5	5	2	-	-	-	-	-	5	[87]
JN1	43	24	18	5	-	5	5	-	-	-	-	-	-	-	[44]
JN2	43	15	18	-	-	2	10	5	-	-	5	-	-	2	[51]
JN3	51	15	18	-	-	2	2	5	-	-	5	-	-	2	[51]
JN4	45	20	20	-	-	2	2	6	-	-	-	-	-	5	[51]
JZ3	41.8	12.4	17.7	4.7	-	1	5.2	-	6	-	2.7	-	4.8	3.7	[33]
JZ3+	38.7	12.4	19.7	4.6	-	0.8	5.2	-	5.7	-	2.3	-	4.9	5.7	[33]
JZ4 *	38.9	12.5	17.8	5	-	1.1	5.2	6.2	-	-	2.3	-	5.2	5.8	[34]
JZ5 *	32	20.4	19.2	4.5	-	0.9	4.7	6.3	-	-	1.1	-	5.2	5.7	[34]
KZ4	53	24	18	-	-	-	5	-	-	-	-	-	-	-	[88]
KZ5	48	24	18	5	-	-	5	-	-	-	-	-	-	-	[88]
KZ6	42	24	18	5	--	-	5	-	6	-	-	-	-	-	[89]
KZ7	53	24	18	5	-	-	-	-	-	-	-	-	-	-	[88]
OHS1	38	24	18	5	-	-	5	-	-	-	-	-	5	5	[32]
NV1	53	23	5	5	-	5	2	-	-	5	-	-	-	2	[16]
NV2	43	30	10	2	-	2	5	-	-	-	-	3	-	5	[13]
NV5	43	24	18	-	-	-	5	-	-	-	-	5	-	5	[11]
TT1	50	24	18	-	8	-	-	-	-	-	-	-	-	-	[90]
TT2	48	24	16	-	7	-	5	-	-	-	-	-	-	-	[36]
TT3	48	24	16	5	7	-	-	-	-	-	-	-	-	-	[36]
TT4 *	42.4	24.6	15.7	5	6.9	-	5.4	-	-	-	-	-	-	-	[36]
TT5	37	24	18	5	5	-	5	-	6	-	-	-	-	-	[35]
TT6	39	24	18	4	6	-	5	-	-	-	-	-	-	4	[35]
TT7	38	24	17	5	6	5	5	-	-	-	-	-	-	-	[35]
TT8	42.5	24	17	3.5	6	5	2	-	-	-	-	-	-	-	[36]
YG8	67	-	20	-	-	5	-	5	-	-	3	-	-	-	[91]
YG10	59	10	18	-	-	5	-	5	-	-	3	-	-	-	[92]
YG11	54	10	18	5	-	5	-	5	-	-	3	-	-	-	[92]
ZF6	43	24	18	5	-	-	5	-	-	-	-	-	5	-	[93]
ZF9	38	24	18	5	-	5	5	-	-	-	-	-	5	-	[93]
ZX5	51	24	18	5	-	-	-	-	-	-	-	-	-	2	[17]
ZX7	46	24	18	5	-	-	5	-	-	-	-	-	-	2	[17]

* actual composition.
